# The Hinge Region of Human Thyroid-Stimulating Hormone (TSH) Receptor Operates as a Tunable Switch between Hormone Binding and Receptor Activation

**DOI:** 10.1371/journal.pone.0040291

**Published:** 2012-07-06

**Authors:** Ritankar Majumdar, Rajan R. Dighe

**Affiliations:** Department of Molecular Reproduction, Development and Genetics,Indian Institute of Science, Bangalore, Karnataka, India; University of Cambridge, United Kingdom

## Abstract

The mechanism by which the hinge regions of glycoprotein hormone receptors couple hormone binding to activation of downstream effecters is not clearly understood. In the present study, agonistic (311.62) and antagonistic (311.87) monoclonal antibodies (MAbs) directed against the TSH receptor extracellular domain were used to elucidate role of the hinge region in receptor activation. MAb 311.62 which identifies the LRR/Cb-2 junction (aa 265–275), increased the affinity of TSHR for the hormone while concomitantly decreasing its efficacy, whereas MAb 311.87 recognizing LRR 7–9 (aa 201–259) acted as a non-competitive inhibitor of Thyroid stimulating hormone (TSH) binding. Binding of MAbs was sensitive to the conformational changes caused by the activating and inactivating mutations and exhibited differential effects on hormone binding and response of these mutants. By studying the effects of these MAbs on truncation and chimeric mutants of thyroid stimulating hormone receptor (TSHR), this study confirms the tethered inverse agonistic role played by the hinge region and maps the interactions between TSHR hinge region and exoloops responsible for maintenance of the receptor in its basal state. Mechanistic studies on the antibody-receptor interactions suggest that MAb 311.87 is an allosteric insurmountable antagonist and inhibits initiation of the hormone induced conformational changes in the hinge region, whereas MAb 311.62 acts as a partial agonist that recognizes a conformational epitope critical for coupling of hormone binding to receptor activation. The hinge region, probably in close proximity with the α-subunit in the hormone-receptor complex, acts as a tunable switch between hormone binding and receptor activation.

## Introduction

The Glycoprotein hormone receptors (GpHR) comprising of TSHR, LHR and FSHR are GPCRs with rhodopsin type transmembrane domain (TMD) and comparatively large extracellular domains (ECD). The crystal structures of FSHR-ECD (G17-S268) co-crystallized with a single chain FSH [1] and TSHR ECD (M22-L260) with the stimulatory F_AB_ M22 [2] reveal that the N-terminal ECD comprises of leucine rich repeats (LRRs) in a scythe blade arrangement of β-loop-β motifs. The heterodimeric hormones (FSH, LH, hCG and TSH) comprising of an identical hormone α-subunit annealed non-covalently with the hormone specific β-subunit interact with the concave portion of the LRRs. The GpHR activation requires the signal generated by binding of the hormone at LRRs to be transmitted to the intracellular signaling component via TMDs, a mechanism not well understood.

One of the receptor activation models (scaffold model) suggests that the C-terminal region of ECD, which links LRRs to TMD, acts as a flexible “hinge”, enabling the hormone bound LRR to communicate with the Extracellular loops (ECL) of TMD [3]. The scaffold model, however, does not explain the cryptic hormone binding sites in the hinge region of FSHR [4] or the interaction of the α-subunit of hormones with the hinge regions [5]. Removal of ECD by trypsin treatment [6], or mutations or deletions [7] in the hinge region cause an increase in hormone-independent activation by releasing the silencing effect of the ECD and have since given rise to the tethered inverse agonist model in which ECD serves as an inverse agonist stabilizing TMD in an inactive conformation [8]. Hormone binding or activating mutations in the hinge region disengages these inhibitory ECD-TMD interactions. However, this model is inadequate to explain relatively low activation of hinge region constitutively activating mutations (CAMs) like K287A or K291A compared to that caused by the hormone even with ample expression of these mutants on cell surface [9]. It also does not explain lack of hormone-independent activation of LHR [10] or why S277Q in LHR responds to hCG but TSHR homologous mutation S281Q does not respond to TSH, although certain mutations in the critical S281 residue has the potential of stimulating TSHR comparable to the hormone [11].

Though gain or loss-of-function mutations in GpHRs has been a popular approach to understand the molecular details of receptor activation, its major limitation include receptor misfolding, irreversibility, lack of plasma membrane translocation and constraining of the receptor into a non-natural conformational states. In contrast, antibodies, both polyclonal and monoclonal, have exquisite conformational specificity and extensively used in hormone-receptor interactions studies [12]. Our earlier studies using agonistic FSHR hinge antibody [13] as well as extensive use of polyclonal and monoclonal antibodies in investigation of structure-function relationship of TSHR and its ligands [14] clearly indicate the expediency of using such antibodies in studying the role of hinge region in receptor activation. In the present study, we have combined mutagenesis and novel TSHR MAbs to dissect out roles of different regions of the receptor in binding and signaling.

## Materials and Methods

### Stable Cell Line Expressing hTSHR

HEK 293 cells (*ATCC*-CRL-1573; American Type Culture Collection) were transfected with the full-length pcDNA3.1-hTSHR cDNA (Missouri S&T cDNA Resource Center, USA) and the stable clones expressing hTSHR were selected on G-418 (1 mg/ml) and further characterized for their ability to specifically bind ^125^I-human TSH (hTSH)/bovine TSH (bTSH) with high affinity and exhibiting cAMP production in response to the hormones.

### Exodomain Fragments of TSH Receptor

Different overlapping fragments encompassing the entire TSH receptor ECD were cloned, expressed in E. coli as GST fusion proteins and purified from the lysates (Figure S1A). These include 1] the first three LRRs (TLRR 1–3, amino acid (aa) 21–127), 2] the first six LRRs (TLRR 1–6, aa 21–200), 3] the putative major hormone binding domain (TLRR 4–6, aa 128–200), and 4] the hinge region of TSH receptor along with LRR 7 to 9, (TLRR 7-HinR, aa 201–413). The receptor fragment TLRR 7-HinR was further subdivided into LRR 7–9 (TLRR 7–9, aa 201-161) and the hinge region (TSHR HinR, aa 261–413), expressed as N-terminal His-Tagged protein and purified using IMAC chromatography (Ni-Sepharose, GE Biosciences). The full length TSHR-ECD, (aa 21–413) was expressed using the *Pichia pastoris* expression system and purified from the fermentation supernatant using hydrophobic interaction chromatography followed by Sephacryl S200 size exclusion chromatography Purity of each receptor fragment was ascertained by SDS-PAGE and western blotting analysis using antibodies against their respective protein tags (GST/His) (Figure S1B). The identity and purity of TSHR-ECD protein were demonstrated by western blot analysis using a TSHR polyclonal antibody as described previously [15].

### Receptor Antibodies

MAbs against TLRR 7-HinR fragment and TSHR-ECD were developed according to the protocol described previously [16]. MAbs were screened for binding to TSHR-ECD and five MAbs purified from the medium or ascites by Protein-G chromatography, were further characterized for binding to TSHR by immunocytochemistry and flow cytometry. F_Ab_ fragments were generated from the IgG by papain digestion and purified using Protein G chromatography. MAb 2A6 was developed against hFSH and was shown to bind to all three Glycoprotein hormones indicating hormone α-subunit specific epitope (Majumdar and Dighe, unpublished data). Polyclonal antibodies were raised against the receptor fragment TLRR 1–6 and TSHR hinge region using protocols described earlier [17].

### Epitope Mapping by Peptide Phage Display Analysis

The epitope of one of the MAbs was identified using the Ph.D.-12 Phage Display Peptide Library (New England BioLabs Inc.) comprising of a random dodecapeptide fused at the N-terminus of the minor coat protein III of M13 phage. Three rounds of biopanning were carried out according to the manufacturer’s instructions with some modifications. Briefly, 2×10^11^ phages were allowed to bind to immobilized purified MAb. The unbound phages were removed by increasingly stringent washes and finally eluted with 100 µl of elution buffer (0.2 M glycine–HCl, pH 2.2, containing 1 mg/ml BSA) for 10 minutes at room temperature and immediately neutralized with 15 µl of 1 M Tris–HCl (pH 9.1). The eluted phages were reamplified, titrated and used for subsequent rounds of panning with 40 and 10 µg/ml of MAb in the second and third rounds respectively and used for DNA sequencing and immuno-analysis. The sequences of the dodecapeptides appearing more than five times in the selected phage clones were classified as the consensus sequence. The resultant sequence was scanned against the full length TSH receptor with non-stringent gap opening and extension penalties (5 and 20 respectively in Clustal 2.0.12 software).

### TSHR Truncations and Mutations

Various deletions and truncations were introduced into TSHR and cloned into a modified pcDNA3.1- MycHis vector (Invitrogen) containing a 63 nucleotide fragment encoding the TSHR cognate signal peptide at the Hind III-Eco RI sites. DNA fragments encoding hTSHR Hinge TMD (TSHR HinTMD, aa 261–764) and only TMD (TSHR TMD, aa 414–764) were cloned downstream of the signal sequence to enable proper translocation to cell surface. The hinge region activating mutations S281I, gain-of-function mutation in TSHR ECL 1/2/3 - I486F, I568T and V656F, and inactivating mutation D410N were introduced into TSHR wild type background using a two-step PCR based mutagenesis [18]. Double mutants SL266.267RE and TR273.274LE were created similarly, which were then used as template to assemble the quadruple mutant SL266.267RE/TR273.274LE. The constructs, TSHR E251K, TSHR-LHR-6 and TSHR-LHR-6A1 were the kind gifts of Prof. Basil Rapoport, UCLA, USA.

### Transfection Experiments

HEK 293 cells were seeded into 6-well (∼10^6^ cells/well/2 ml), 24-well (∼3×10^5^ cells/well/500 µl) or 48-well (∼10^5^ cells/well/250 µl) tissue culture plates in DMEM supplemented with 10% fetal bovine serum and grown to obtain nearly 80–90% confluency. These were transiently transfected with different recombinant hTSHR constructs (3.2 µg of the plasmid DNA/ml of the plating medium) using Lipofectamine 2000 reagent as per the manufacturer’s protocol (Invitrogen) and transgene expression studies were carried out 48 h later. In each experiment, parallel plates were transfected simultaneously to determine ligand binding to the intact cells and membrane preparations, Flow cytometric analysis, cAMP production, and Western blot analysis.

#### Receptor Binding

Radioiodination of bTSH/hTSH was carried out using the IODO-GEN method [19]. Binding characteristics of the wild type and mutant TSH receptors were determined by radioreceptor assay by incubating the membrane preparation (10 µg) with different concentrations of hTSH/bTSH in presence of 0.14 nM of ^125^I-hTSH/bTSH (specific activity of the tracer −0.26 µCi/fmol) at 37°C for 2 h in a reaction volume of 250 µl. The receptor bound radioactivity was centrifugally separated (4000 g at 4°C for 20 minutes) after addition of 2.5% PEG 6000 and counted in Perkin Elmer γ-counter. The non-specific binding was determined in presence of excess unlabeled hTSH or bTSH for their respective tracer (1 µg/ml). The binding data were analyzed using non-linear regression analysis and affinity (*K_d_*) of hTSH and *B_max_* calculated by fitting the binding data into the equation
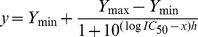
where y is specifically bound hTSH, *x* is the concentration of unlabeled hTSH added, h is the Hill slope of the regression *and Y_max_*, *Y_min_* are the upper and lower plateaus of the the regression respectively. *K_d_* was calculated using the Cheng-Prussof equation,



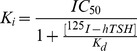
and which for homologous tracer reduces to, 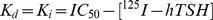. *B_max_* was calculated by using equation,



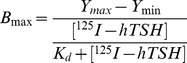
where [*^125^I(hTSH)*] is the concentration of ^125^I-TSH used and *K_d_* is the affinity of hTSH calculated as mentioned. All curve fitting was carried out using GraphPad prism 5.0. The binding data were converted to Scatchard plot for visual representation. Ability of various antibodies (IgGs) to inhibit ^125^I-hTSH binding was investigated by incubating the receptor preparations with antibodies prior to addition of ^125^I-hTSH and the mechanism of inhibition was analyzed as discussed in the result section. Binding of ^125^I-MAb/F_AB_ fragments to HEK293-TSHR was performed similar to those carried out with radiolabelled hormone the details of which have been furnished in the legends of the Figure S9.

### In Vitro Bioassay and cAMP Measurement

Approximately10^5^ cells/well (stable cell line or transiently transfected) were plated in a 48-well plate and 24 h later incubated with fresh medium containing 1 mM phosphodiesterase inhibitor, 3-isobutyl-1-methylxanthine for 30 min at 37°C in a CO_2_ incubator (100 µl). The cells were then exposed to varying concentrations of hTSH or TSHR polyclonal or monoclonal IgGs for either 15 min or 1 h respectively at 37°C (100 µl). The cells were lysed with addition of 200 µl of 0.2 N HCl, and total cAMP produced was determined by cAMP RIA as described elsewhere [15].

### Flow Cytometric Analysis of TSHR Mutants

Flow cytometric analysis was performed to quantify the cell surface expression of different TSHR mutants and identify/locate the putative epitopes recognized by TSHR MAbs. The cells transfected with different TSHR constructs were detached by treatments with Ca^+2^/Mg^+2^ free PBS containing 1 mM EDTA and EGTA and washed with PBS followed by incubation with different TSHR MAbs in PBS containing 5% FBS at 4°C for 1 hour. The cells were washed twice with the same buffer and incubated at 4°C for 1 h with a 1∶500 dilution of FITC-conjugated secondary antibody (Sigma Chemical Co, USA) and the cell surface binding of the MAbs was assessed using the FACSCANTO II (Becton-Dickinson, Franklin Lakes, NJ, USA), flow-cytometer. The surface expression of various TSHR mutants was ascertained by normalizing the median fluorescence intensity (MFI) with a control antibody as discussed below.

## Results

### Characterization of Human TSHR Cell Line

HEK293-hTSHR cell line exhibited high affinity binding to both hTSH/bTSH with affinity for bTSH (2.4×10^−10^ M) being higher than that for hTSH (5.3×10^−10^ M) as reported previously [20] while the number of binding sites for both hormones was found to be similar (∼3.45 pmols/mg of membrane protein) (Figure S2, *Inset*). The cell line responded to both hTSH and bTSH with a dose-dependent increase in total cellular cAMP with *EC_50_* of 2.2 and 0.7×10^−10^ M respectively (Figure S2).

### Binding Specificity and Characterization of TSHR Exodomain Antibodies

Polyclonal antibodies against TLRR 1–6 and TSHR HinR receptor fragments were characterized for their binding to different TSH receptor fragments (Figure S3A) and their ability to recognize the full length TSH receptor (Figure S3B). Both polyclonal antibodies exhibited binding towards full length ECD, as well as, their cognate antigenic proteins, but exhibited little or no cross reactivity with other TSH receptor fragments.

**Figure 1:**
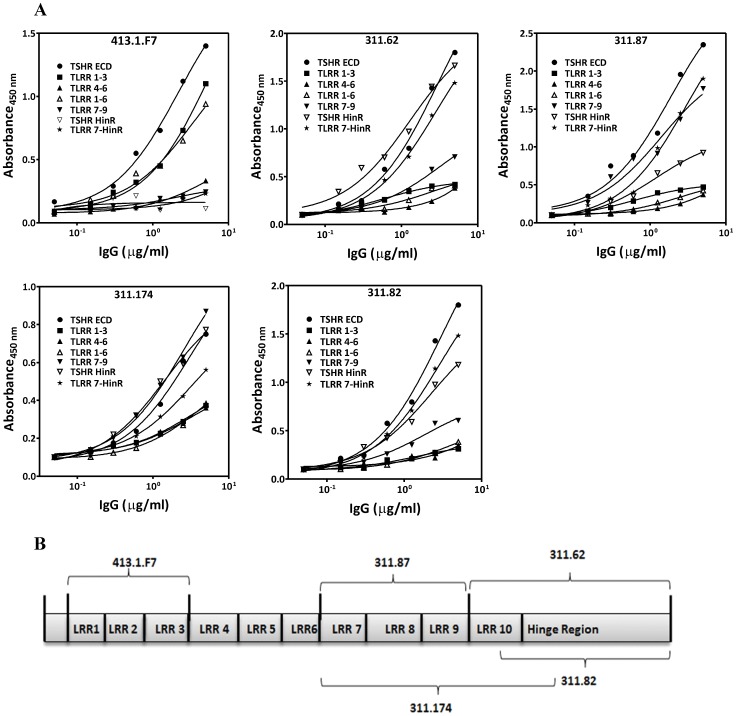
Putative epitope mapping of TSHR MAbs using TSH receptor fragments. **A**. Various fragments of TSHR (50 ng/well) were adsorbed on to a plastic surface and incubated with 100 µl of increasing concentrations of IgG from each TSHR MAb followed by addition of goat anti-mouse IgG-peroxidase and determination the enzyme activity. **B**. Schematic diagram of putative binding regions of each TSHR MAb used in this study.

Five monoclonal antibodies, developed against TLRR 7-HinR or TSHR ECD exhibiting high affinity binding to HEK293-hTSHR in flow cytometric or immunochemical analysis (Figures S4 & S5), were chosen to investigate effects on hormone–receptor interactions after ascertaining their monoclonality (Table S1). Analysis of the putative binding domains of these MAbs based on ELISA (Figure 1A) with different TSHR fragments indicated that MAb 413.1.F7 recognizes TLRR 1–3, MAb 311.87 recognizes TLRR 7–9, MAb 311.62 recognizes the last LRR with a major binding site in the hinge region while MAb 311.82 is a hinge region specific antibody. The epitope recognized by the MAb 311.174 spans mostly the LRR 7–10 (Figure 1B).

### Effect of TSHR ECD Antibodies on Hormone-receptor Interactions

Ability of TSHR MAbs to influence the functionality of hTSHR was investigated by determining their effects on hormone binding and the basal and hormone-stimulated cAMP production.

#### Hormone binding

**Figure 2:**
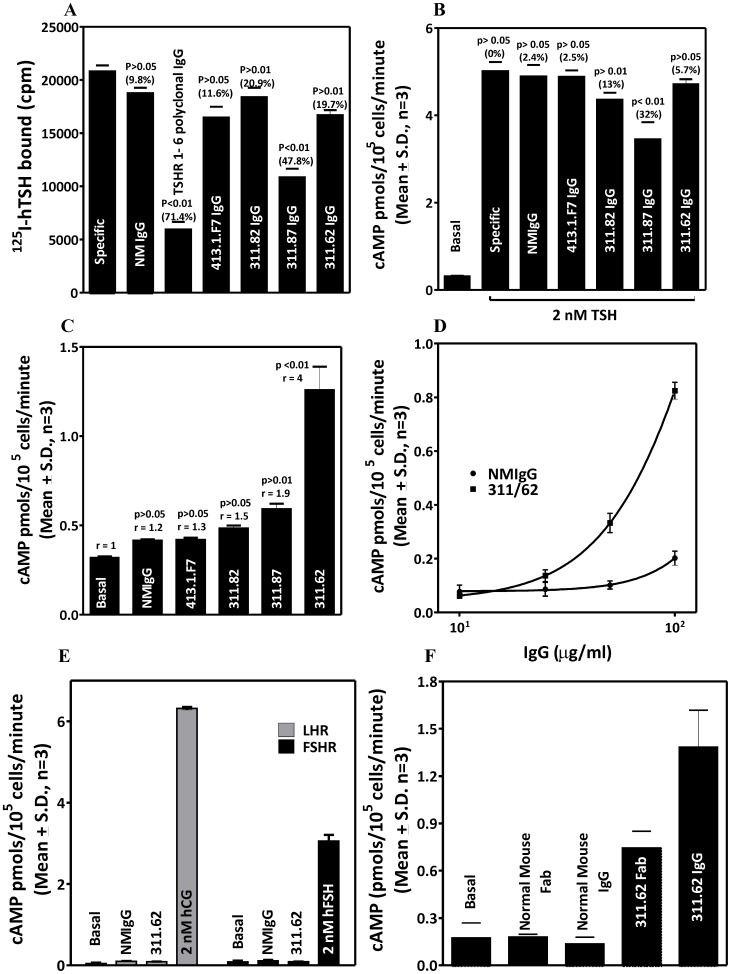
Effect TSHR MAbs on TSH-TSHR interactions. **A**. Membrane preparation from HEK293-hTSHR was incubated with different TSHR MAb (50 µg IgG/ml) for 1 h at room temperature followed by incubation with ^125^I-hTSH and the bound hormone was determined. NMIgG and TLRR1-6 IgG were used as negative and positive control respectively. **B.** HEK293-hTSHR cells were incubated with TSHR MAbs (50 µg IgG/ml) in the presence of hTSH (2 nM) for 15 min at 37°C, and cAMP produced was determined by RIA. In all of experiments, the basal cAMP production was determined without hTSH or antibodies. The bracketed *numbers above the bars* denote percentage of inhibition of hTSH binding or stimulation in the presence of antibodies and the statistical significance of the inhibition for each MAb as compared to the control is denoted by the *P-value* calculated from the two-tailed unpaired t-test. **C.** HEK293-hTSHR cells were incubated with IgGs of TSHR MAbs (125 µg/ml) in the absence of hTSH for 1 h at 37°C, and cAMP produced determined by RIA. The *r values above* the *bars* denote fold increase in cAMP production in presence of antibodies over the basal level and the p-value the statistical significance. **D**. HEK293-hTSHR cells were incubated with increasing concentrations of NMIgG, or MAb 311.62 IgG for 1 h at 37°. **E**. HEK293 cells expressing hLHR and hFSHR were incubated with NMIgG/311.62 IgG or their respective hormones. **F**. HEK293-hTSHR cells were incubated with NMIgG/F_AB_ or MAb 311.62/F_AB_ (125 µg/ml) for 1 h at 37°C. In all experiments, cAMP produced was quantified by RIA. All of the experiments presented above are representative of at least two independent experiments.

Membrane preparation from HEK293-hTSHR cells was pre-incubated with either Normal Mouse IgG (NMIgG) or TSHR MAbs for 1 h at 37°C, followed by incubation with ^125^I-hTSH for an additional 1 h and determining the bound hormone. As shown in Figure 2A, the polyclonal antibody TLRR1-6 inhibited hormone binding by ∼75%. Of the three MAbs tested, only MAb 311.87 showed significant (∼45%) inhibition of hormone binding while inhibition exhibited by other MAbs was marginal.

#### cAMP production

HEK293-hTSHR cells were first incubated with antibodies for 1 h, followed by addition of buffer or hormone and the total cAMP formed at the end of 15 minutes incubation was estimated. As shown in Figure 2B, the pattern of inhibition of hormone response by the antibodies was similar to the effect on binding. The MAb 311.87 inhibited both binding and response to nearly the same extent while the others did not have any effect on hormone stimulated response.

Interestingly, only MAb 311.62 by itself increased cAMP levels in the absence of the hormone in a dose dependent manner *(* Figures 2C&D *)* with nearly 5–8 fold increase in cAMP levels with 100 µg/ml of the antibody suggesting the stimulatory nature of this MAb. This agonistic behavior of MAb 311.62 was TSHR specific as it had no effect on hLHR or hFSHR expressing cell lines (Figure 2E). In Flow cytometric analysis, this MAb showed little cross-reactivity with LHR (5–10%), but not with FSHR (Figure S6). The F_AB_ fragment prepared from this MAb could also stimulate cAMP production in hTSHR cells (Figure 2F) in a dose dependent manner (Figure S8) suggesting that antibody induced receptor dimerization may not be the cause of stimulation. This observation is further supported by FRET/BRET observations made in TSHR [21] and LHR [22] where no correlation between hormone stimulation and receptor dimerization was reported.

### Effect of TSHR MAbs on the affinity of hTSHR for hTSH

**Figure 3:**
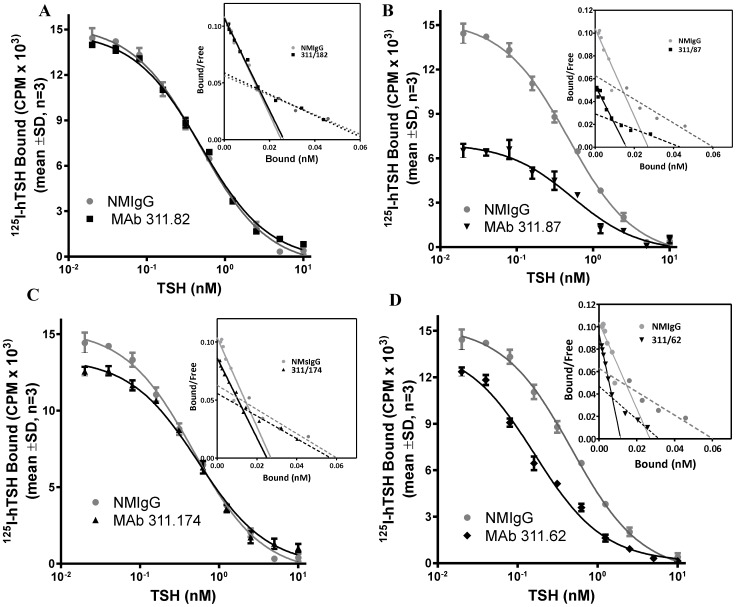
Effect of TSHR MAbs on the affinity of TSHR for TSH. ^125^I-hTSH was incubated with hTSHR membranes (20 µg/ml) with increasing concentrations of the unlabelled hTSH in the absence or presence of 50 µg/ml of A. 311.82 IgG, B. 311.87 IgG, C. 311.174 IgG or D. 311.62 IgG and affinity (*K_d_*) of hTSH and *B_max_* calculated as described in the materials and methods section.

Effect of MAbs on hTSH-TSHR interactions was further investigated by determining binding constants in presence of MAbs. Typical TSH radioreceptor assays (RRA) were carried out after pre-incubating the receptor preparation with MAbs or NMIgG and the binding data was converted to the Scatchard plots and the binding constants calculated according to the legends of Figure 3. The Scatchard analysis of TSH-TSHR binding is typically curvilinear and for visual representation, only the physiologically relevant high affinity receptor component has been considered. The effect of the antibodies on the low affinity, high capacity components is, however, presented in Figure S10. As shown in Figure 3 and Table 1, MAbs 311.82 and 311.174 did not affect the TSH-receptor interactions, although a marginal decrease in the *B_max_* was observed in case of the latter MAb. The MAb 311.87 exhibited non-competitive inhibition with a significant decrease in *B_max_* and no change in the affinity.

**Table 1:** Determination of affinity of TSHR for TSH in presence of TSHR MAbs.

Antibody	*K_d_* (nM)	*Bmax* (nM)
No IgG added	0.259±0.013	0.028±0.0031
NMIgG	0.29±0.033	0.027±0.0012
311.82	0.30±0.041	0.027±0.0081
311.174	0.34±0.051^b^	0.024±0.011
311.87	0.39±0.081^b^	0.011±0.0011^a^
311.62	0.10±0.066^a^	0.019±0.0051^a^

The membrane preparation (20 µg/ml) obtained from HEK293-hTSHR cells were incubated with either 50 µg/ml of NMIgG or TSHR MAbs. ^125^I-hTSH (2×10^5^ cpm) was added to TSHR MAb –TSH complex in presence of increasing concentration of hTSH and *K_d_* and *B_max_* value obtained by fitting the data as mentioned in the legends of Figure 3.

a p<0.01 versus values with no IgG added. The values are expressed as the mean ± S.D. of triplicate determinations.

b p<0.05 versus values with No IgG added.

Surprisingly, although MAb 311.62 did not exhibit any effect on hormone–receptor binding (as shown by insignificant change in the Bound/Free ratio in the Scatchard plot), a significant increase in the affinity for hTSH was observed in presence of MAb 311.62, with ∼2.5 fold decrease in *K_d_*.

### Identification of the Stimulatory MAb Binding Site

**Figure 4:**
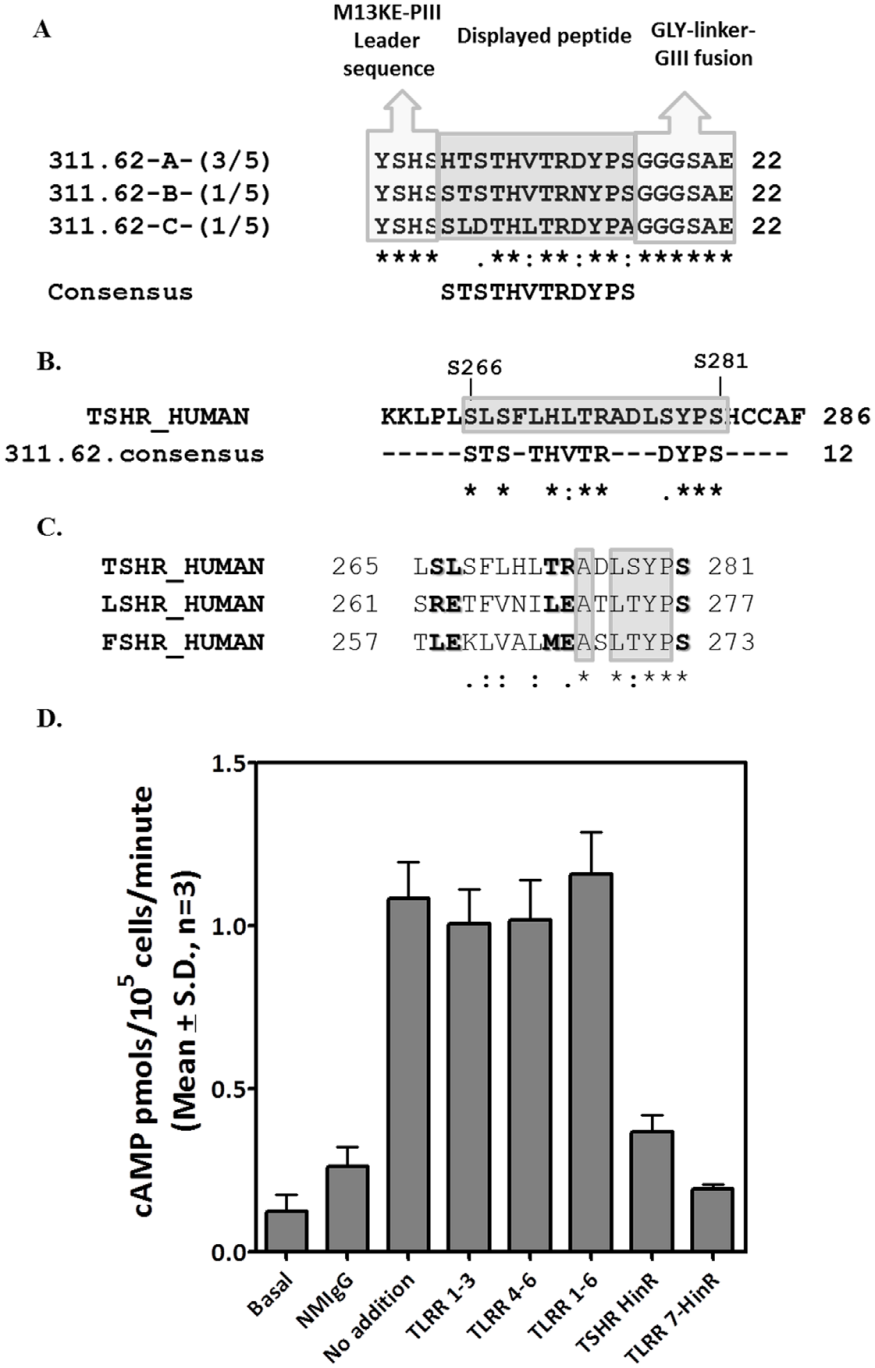
Epitope mapping of MAb 311.62 by Peptide Phage Display analysis. **A**. Amino acid sequences deduced from the phage clones selected after three rounds of biopanning. Three groups of sequence A, B, C were obtained respectively on analyzing the sequence and consensus obtained for each. The numbers in the parentheses denote the frequency or ratio of the number of the phage clones expressing the common peptide sequence to that of the total phage clones. **B**. Sequence alignment of the consensus sequence derived from the phage clones with the full length TSH receptor revealed sequence similarity in TSH receptor region 266–281. **C**. Multiple sequence alignment of hTSHR, hLHR and hFSHR corresponding to the region 265–281 of hTSHR. TSHR residues marked in bold were mutated to corresponding residues of LHR. D. MAb 311.62 IgG (25 µg/ml) was pre-incubated with different TSH receptor fragment protein (50 µg/ml) and then added to HEK293-hTSHR cells, and cAMP produced was determined.

Ability of different receptor fragments to block antibody stimulated response was used to locate the epitope of the MAb. As shown in the Figure 4D, pre-incubation of the antibody with TLRR7-HinR protein and hinge region inhibited antibody stimulated response indicating that the epitope resided in the hinge region of the receptor. This was further confirmed by screening the peptide phage-display library with the purified 311.62 IgG. Comparison between the consensus amino acid sequence derived from these phages, (Figure 4A) and TSHR revealed that aa 265–281 possessed 67% sequence identity (83% sequence similarity) and was one of the potential candidates in discontinuous epitope (Figure 4B). This part of TSHR is highly variable in GpHRs, thus accounting for the specificity of the stimulatory antibody (Figure 4C).

### Analysis of MAb 311.62/MAb 311.87 Binding to TSHR Hinge Region and ECL Mutants

Rapoport and co-workers reported that replacement of hinge region residues, aa 261–316 [23] or 270–278 [24] with the corresponding residues from LH receptor increased affinity of the receptor for the hormones. Perceptible increase in the affinity was also observed in the Cysteine box-2 (Cb-2) constitutively activating mutation (CAM) S281I [25] and S281HCS [26] and the corresponding LHR mutation S277I [27]. As these residues form the binding pocket of the MAb 311.62 and exhibited similar increase in hormone affinity, it was interesting to study binding of MAb 311.62 to these mutants.

Flow cytometric analysis of binding of MAbs 311.62 and 311.87 to different TSHR mutants was carried out to determine differential binding among various TSHR mutants. The median fluorescence intensity of each antibody was normalized to NMIgG and expressed as relative median fluorescence intensity (RMFI). RMFI for each mutant was compared to that of that of the wild type (RMFI_MUT_/RMFI_WT_) to provide a semi-quantitative estimation of the degree of binding of a given TSHR MAb to a TSHR mutant. Further, relative surface expression (*R_e_*) of the mutant receptors was calculated by comparing binding of MAbs to that of a control antibody such as TLRR 1–6 antibody or TSHR MAb 413.1.F7 which recognized the epitopes in the N-terminal region of TSHR distal from the mutagenic sites. *R_e_* values derived from both these antibodies were comparable for any given mutant validating the choice of control antibodies. RMFI_MUT_/RMFI_WT_ values for MAb 311.62 or MAb 311.87 having 2 S.D from *R_e_* of a mutant was considered loss or gain of recognition to that construct.

#### Binding of MAb 311.62 to TSHR mutants

As shown in *Table 2*, replacement of TSHR residues 273 and 274 by the corresponding amino acids of LH receptor (TR273.274LE) abrogated MAb 311.62 binding while surface expression (*R_e_*) of the mutant receptor was 87% of the wild type. Similar mutations at 266 and 267 residue (SL266.267RE) decreased MAb 311.62 binding to 60% over the *R_e_* value for this mutant. Surface expression for the quadruple mutant SL266.267RE/TR273.274LE was <20% of the WT as indicated by its *R_e_* value with no binding observed for MAb 311.62. The decreased MAb binding to the mutant S281I, but not to the loss of function mutant D410N, confirmed the data obtained from the peptide phage display. It is also interesting to note that the critical residues T273 and R274 are also part of the epitope of MAb CS-17, an inverse agonistic antibody. Loss of MAb 311.62 binding to TSH-LHR-6 or TSH-LHR-6A1 chimeric constructs (*Table 3*), where the hinge of TSHR has been replaced by that of LHR but not to the LRR deleted construct (TSHR HinTMD) indicate that MAb 311.62, unlike CS-17 [28] has no additional epitopic site in the LRR and thus may explain their differential action.

**Table 2:** Surface expression of TSHR mutants and their relative binding to MAb 311.87 and MAb 311.62.

	NMIgG	TLRR 1–6 Polyclonal		413.1.F7		311.87		311.62	
Construct	MFI	RMFI‡	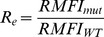	RMFI‡	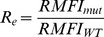	RMFI‡	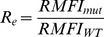	RMFI‡	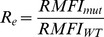
pCDNA3.1	2.64±0.12	1.29±0.43	NS	0.83±0.22	NS	1.33±0.26	NS	1.65±0.11	NS
WT	4.22±0.31	65.8±8.34	1	28.55±3.45	1	32.2±6.55	1	60.03±9.22	1
S281I	3.82±0.42	22.7±2.61	0.34	11.7±2.87	0.4	8.37±1.33	0.26^c^	8.7±1.5	0.14^b^
mE251K	2.91±0.33	46.7±4.32	0.71	17.8±4.32	0.63	16.5±3.65	0.51^c^	10.8±2.67	0.18^a^
SL266.267RE	3.78±0.62	49.65±3.25	0.75	22.84±3.22	0.8	15.46±4.32	0.48^b^	35.4±3.76	0.59^c^
TR273.274LE	2.31±0.20	56.5±5.92	0.85	25.9±4.11	0.91	26.4±6.28	0.82	7.02±2.22	0.12^a^
SL266.267RE/TR273.274LE	4.42±0.54	12.5±0.61	0.18	5.95±0.88	0.23	3.84±0.13	0.11^c^	4.80±0.45	0.08^b^
D410N	2.44±0.31	49.35±6.32	0.75	20.32±7.21	0.71	23.18±4.36	0.72	39.61±3.66	0.66
I486F	2.21	25.42±4.32	0.38	9.1±2.33	0.32	13.52±3.87	0.41^c^	32.41±1.46	0.53^b^
I568T	1.43±0.62	43.54±7.22	0.67	21.98±5.54	0.77	21.89±.82	0.67	39.01±4.32	0.64
V656F	1.11±0.35	59.8±6.35	0.9	24.55±3.54	0.86	30.2±6.25	0.93	50.42±8.25	0.83

‡ A positive RMFI (median fluorescence intensity with test antibody/median fluorescence intensity with NMIgG) was defined to be >2.

a p<0.001 versus values obtained from RMFI of the WT. The values are expressed as the mean ± S.D. of triplicate determinations.

b p<0.01,

c p<0.05 versus values with that of WT. NS- Not significant.

**Table 3:** Surface expression of TSHR wild type, deletion and chimeric constructs and their relative binding to MAb 311.87 and MAb 311.62.

Constructs	NMIgG	TLRR 1–6 Polyclonal		413.1.F7		311.87		311.62	
Attribute	MFI	RMFI^‡^	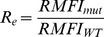	RMFI^‡^	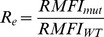	RMFI^‡^	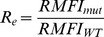	RMFI^‡^	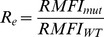
pCDNA3.1	5.16±0.02	1.05±0.11	NS	1.07±0.08	NS	1.45±0.09	NS	1.22±0.21	NS
WT	4.41±0.39	55±5.21	1	31.51±4.53	1	38.4±4.55	1	58.41±2.94	1
TSHR TMD	4.23±0.62	1.4±0.21	NS	1.2±0.14	NS	1.01±0.16	NS	1.23±0.18	NS
TSHR HinTMD	3.0±0.45	4.58±0.45	NS	5.09±0.36	NS	8.06±0.56	0.21	33.94±8.8	0.58
TSHR-LHR-6	4.77±0.35	50.94±6.54	0.92	29.97±7.74	0.94	28.8±3.25	0.75	7.74±0.36	0.13^a^
TSHR-LHR-6A1	5.2±0.48	48.77±9.88	0.88	18.66±8.76	0.59	24.5±3.65	0.63	9.33±9.22	0.16^a^

Symbol legends *vide* Table2.

Aromatic substitutions at Serine 281of TSHR, unlike S281I, lack high constitutive activity [29], perhaps aided cooperatively by the critical residues of the ECLs of TSHR [30]. To investigate how MAb 311.62 binding affects this interaction or whether mutations in ECL changes the MAb epitope, binding of the antibody to the mutants was monitored. Mutations in the second (I568T) and third (V656F) ECLs did not change MAb binding, but increased binding was observed in case of the first ECL mutant I486F (*Table 2*). This could be due to the loss of constraint between Cb-2 and ECLs mediated by S281 and I486 respectively [31] giving rise to a state stabilization of the MAb 311.62 epitope and may serve as direct evidence for spatial reorganization of the TSHR ECD with respect to the ECLs.

MAb 311.62 also failed to recognize the mutant TSHR E251K that binds but does not respond to the hormone, probably due to modification in the flexibility of LRR domain relative to the hinge [32]. Loss of MAb binding may, therefore, be attributed to inaccessibility or change in the conformation of its epitope in this mutant.

#### Binding of MAb 311.87 to TSHR mutants

*The* ELISA data in Figure 1A had indicated that LRR 7–9 (aa 201–260) to be the major epitope recognized by MAb 311.87. This MAb does not recognize TSHR HinTMD that lacks the LRR domain, binds to the chimeric receptors TSH-LHR-6 and TSH-LHR-6A1 but showed relatively low binding to TSHR E251K confirming the identification the epitope. Further, binding of 311.87 was not affected by mutations in the hinge regions or ECLs suggesting LRR 7–9 to be relatively unaffected by any conformational changes occurring downstream to LRRs.

### Differential Effects of MAb 311.62 and MAb 311.87 on Hormone Binding and Response of hTSHR Mutants –TSH-TSHR Mutant Interactions

**Figure 5:**
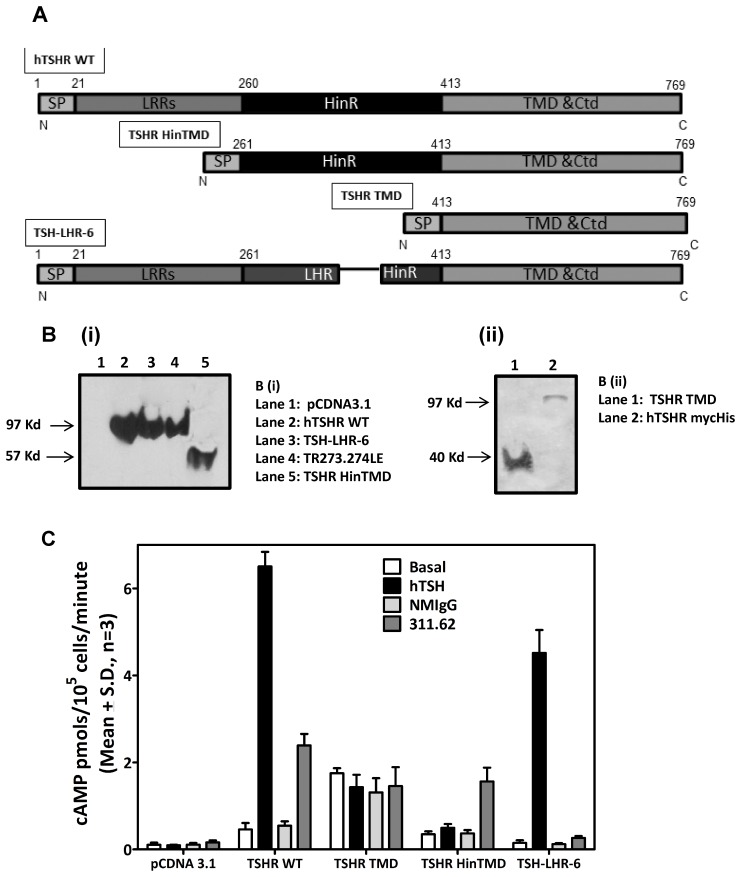
cAMP production by TSHR truncation or chimeric mutants in response to TSH and MAb 311.62. **A**. Schematic representation of hTSHR constructs (not drawn to scale). Soluble plasma membrane preparation from HEK 293 cells, transiently transfected with various TSH receptor constructs were electrophoresed and probed with either (**B(i)**)TSHR HinR specific polyclonal antibody or (**B(ii)**)Anti-His tag monoclonal antibody. A similar full length hTSHR tagged at C-terminal with His-Tag (hTSHRmychis) was created as a positive control. **C**. HEK293 cells transiently transfected with hTSHR constructs were incubated with hTSH (2 nM) for 15 minutes or with MAb 311.62 IgG or NR-IgG (100 µg/ml) for 1 h at 37°C, and cAMP produced was determined. The results presented are representative of two independent experiments.

Affinities of different receptor mutants for the hormone were determined in the absence and presence of MAb 311.62 (Table 4) and MAb 311.87 (Table 5). In the absence of MAbs, all mutants exhibited affinities nearly equal to the wild type hTSHR except S281I, which showed a higher affinity while the quadruple mutant SL266.267RE/TR273.274LE showed lower affinity. The hormone could not bind to the truncated receptors TSHR HinTMD (LRR deleted TSHR mutant, aa 261–764, (Figure 5A)) and TSHR TMD (ECD deleted TSHR mutant, aa 414–764, (Figure 5A)), as both of them lacked the LRRs (Figure 5C). As reported previously [33], TSH-LHR-6 and TSH-LHR-6A1, displayed wild type affinities confirming that the primary hormone-receptor interactions do not involve the hinge region.

**Table 4:** Effect of MAb 311.62 on binding of hTSH to TSHR mutants.

	NMIgG			311.62			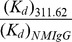
Construct	*K_d_*	*B_max_*	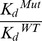	*K_d_*	*B_max_*	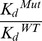	
V.C	ND	ND	–	ND	ND	–	–
WT	0.34±0.029	0.025±0.0015	1	0.17±0.03	0.022±0.0001	1	0.48
S281I	0.20±0.013	0.012±0.0004	0.58	0.16±0.02	0.011±0.005	0.9	0.8
SL266.267RE	0.37±0.028	0.024±0.0004	1.08	0.22±0.01	0.023±0.0004	1.29	0.59
TR273.274LE	0.29±0.026	0.023±0.0006	0.85	0.27±0.06	0.026±0.0008	1.5	0.93
SL266.267RE/TR273.274LE	1.01±0.09	0.017±0.0005	2.97	1.56±0.32	0.009±0.0001	9.17	1.54
D410N	0.32±0.021	0.021±0.0008	0.94	0.16±0.04	0.018±0.0002	0.94	0.50
I486F	0.28±0.021	0.013±0.0005	**0.82**	0.29±0.01	0.011±0.0008	1.7	1.03
I568T	0.38±0.032	0.022±0.0007	1.117	0.33±0.01	0.018±0.0008	1.94	0.86
V656F	0.33±0.01	0.019±0.0006	0.97	0.27±0.02	0.017±0.0002	1.58	0.81

The membrane preparations (10 µg/ml) obtained from different mutants was incubated with either saturating concentrations (340 nM) of either NMIgG or MAb 311.62. ^125^I-hTSH was added to TSHR MAb –TSH complex in presence of increasing concentration of hTSH and *K_d_*_ and_ B_max_ value obtained by fitting the data as mentioned in the legends of Figure. 3.

**Table 5:** Effect of MAb 311.87 on binding of TSH to TSH receptor mutants.

	NMIgG		311.87		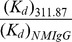	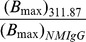
Mutants	*K_d_*	*B_max_*	*K_d_*	*B_max_*	–	–
V.C	ND	ND	ND	ND	–	–
WT	0.32±0.028	0.025±0.0015	0.41±0.03	0.012±0.0012	1.28	0.48
S281I	0.21±0.012	0.012±0.0004	0.31±0.015	0.0053±0.0002	1.48	0.42
D410N	0.35±0.011	0.021±0.0008	0.46±0.021	0.013±0.0006	1.33	0.62
I486F	0.26±0.024	0.013±0.0005	0.37±0.02	0.0063±0.0002	1.42	0.51
V656F	0.33±0.021	0.019±0.0006	0.41±0.036	0.016±0.0009	1.24	0.84
Constructs						
TSHR HinTMD	ND	ND	ND	ND	ND	–
TSH-LHR-6	0.27±0.04	0.022±0.0006	0.39±0.057	0.017±0.0006	1.44	0.77
TSH-LHR-6A1	0.29±0.02	0.019±0.0003	0.35±0.081	0.014±0.0009	1.20	0.73

Membrane preparations obtained from different TSHR mutants were incubated with either NMIgG or MAb 311.87 (134 nM) and *K_d_* or *B_max_* was determined as described in Table 4.

In presence of MAb 311.62, all receptor mutants exhibited increased affinity except the quadruple mutant that showed a reverse effect. However, the effect of antibody was not marked in case of the mutants that showed relatively higher affinity. Interestingly, I468 T (ECL2) V656F (ECL 3) ECL mutants were found to be affected by MAb 311.62 whereas no effect was observed for I486F (ECL 1). This suggests that the epitope of MAb 311.62 is in direct coordination with the first ECL.

In contrast, MAb311.87 showed a marginal decrease in the affinity and *B_max_* for the hormone for all mutants except in case of S281I and V656F. More interestingly, MAb 311.87 decreased the *B_max_* of the TSHR/LHR chimeric constructs similar to that observed with the wild type receptor. This would also suggest that the effect of MAb 311.87 on hormone binding was independent of the hinge region and LRR domain may function as a separate functional unit distinct from the hinge region.

#### Effect of MAbs on the basal and hormone stimulated response by hTSHR mutants

**Figure 6:**
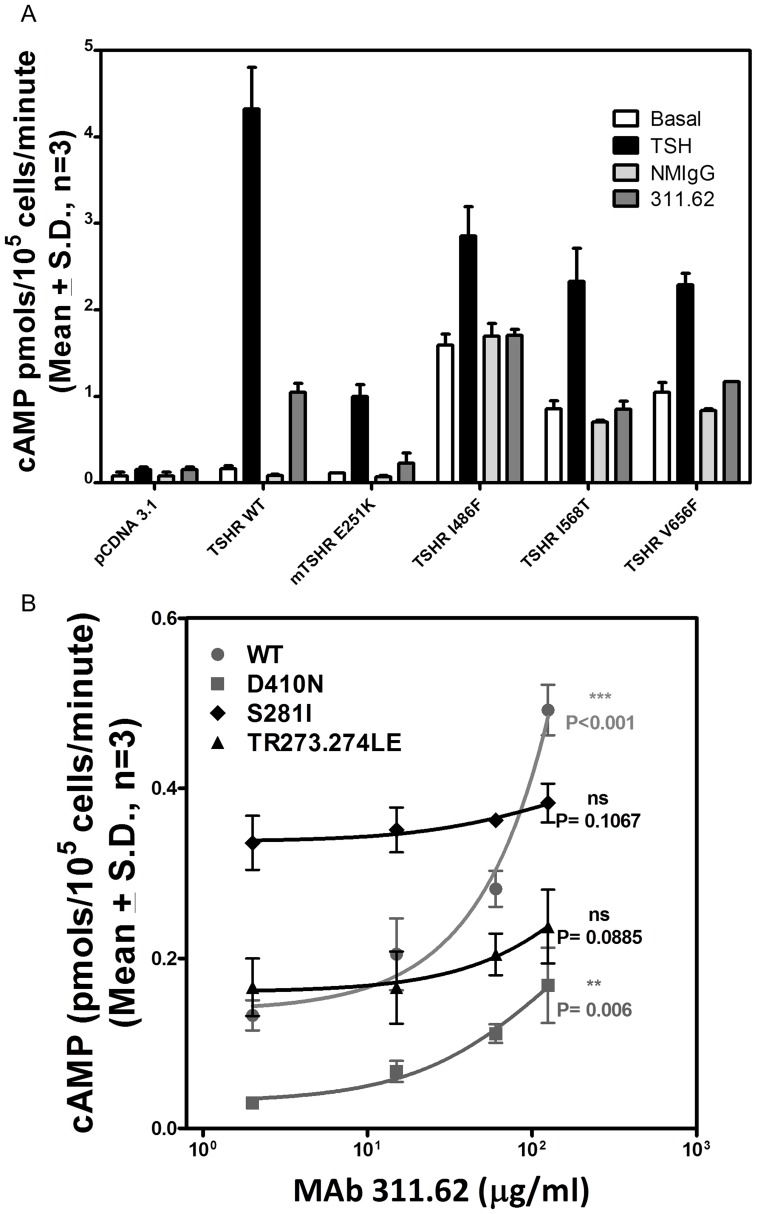
Effect of MAb 311.62 on TSHR mutants. **A**.HEK293-hTSHR cells were transfected with TSHR ECL-CAMs or the inactivating mutation E251K and response to hTSH and MAb 311.62 was determined as described in the legends of Figure 5C. **B**. TSHR wild type and mutants were transiently transfected and cAMP response to increasing concentration of MAb 311.62 was determined. Values besides each cAMP response curve of a given TSHR construct designate the statistical significance of the increase in cAMP produced by the construct in absence of MAb 311.62 and in presence of the highest concentration of MAb 311.62 IgG used in the experiment. ns =  not significant.

The basal and hormone stimulated cAMP produced by the deletion mutants was determined in presence and absence of the antibodies. The cell surface expression of all mutant receptor was confirmed by western blot (Figure 5B) and/or by flow cytometric analysis (Table 3). As expected, TSH did not stimulate deletion mutants lacking LRRs (TSHR TMD and TSHR HinTMD). Complete deletion of the ECD increased the basal cAMP production significantly as reported earlier for TSHR [34] and FSHR [13]. However, presence of hinge region (TSHR HinTMD) not only abolished this increase, but also showed response to MAb 311.62 (Figure 5C). The basal activities of exoloop mutants were much higher than the wild type and could not be stimulated further by the antibody (Figure 6A). While the antibody could not stimulate E251K, it overcame the inactivating effect of D410N and had no effect on the CAM S281I or the TR273.274LE double mutant (Figure 6B).

### Mechanism of Action of TSHR MAb 311.62 and MAb 311.87

Strong negative cooperativity for TSHR has been attributed either to the equilibrium between receptors and receptor-effector complexes with variability in their affinities [35], [36] or to dimerization of TSHR acting “allosterically” through protomer interactions [21]. In either case, ECD of TSHR must go through a substantial conformational change in the hormone binding domain. However, the rigidity of the LRRs suggest that such a change is unlikely without the hinge region playing an important role in it [37]. We investigated effects of MAbs 311.62 and 311.87 on hormone binding to determine whether the Cb-2 region and the LRRs N-terminal to Cb-2 can conformationally alter the hormone binding sites.

**Figure 7:**
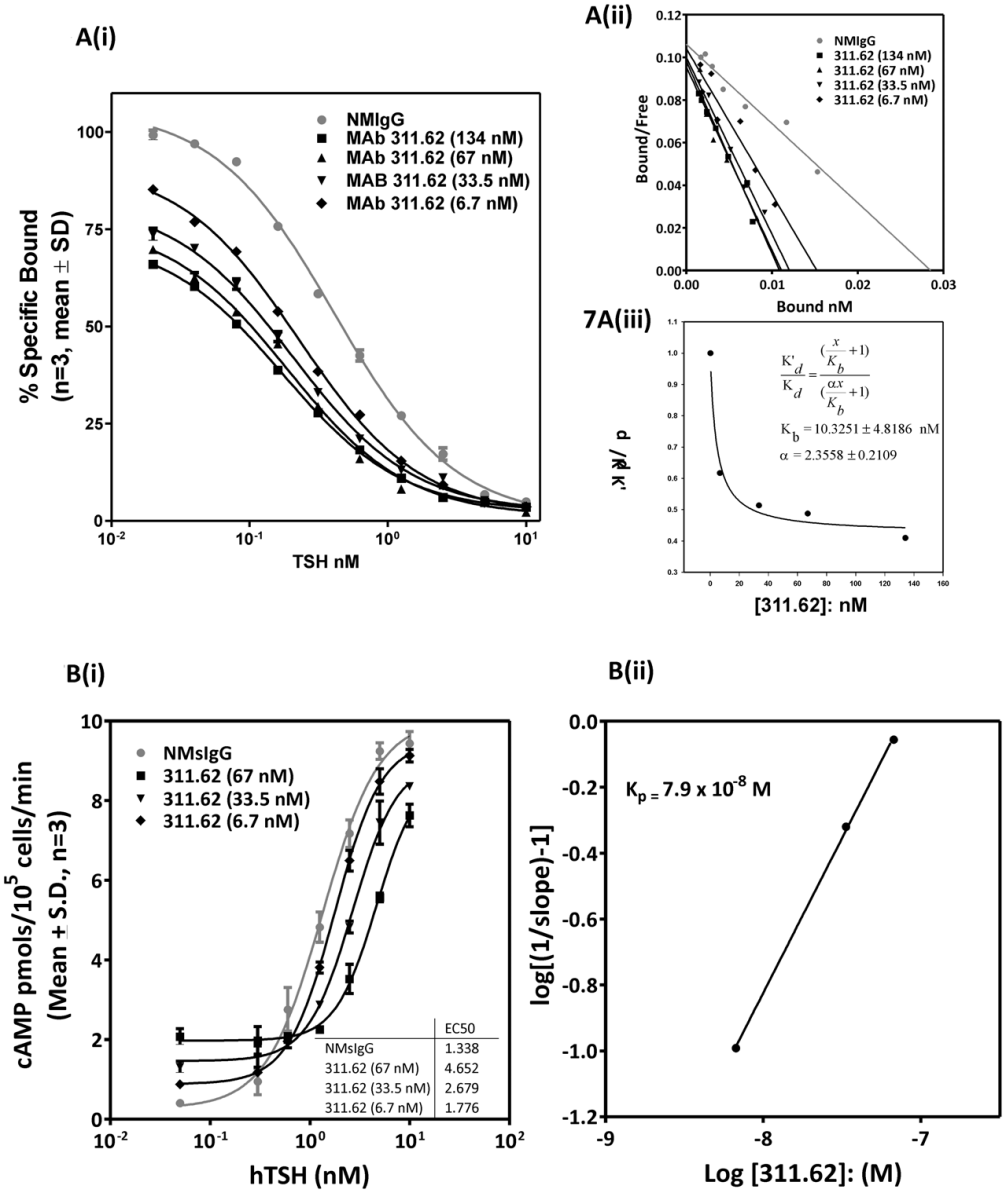
Mechanism of partial agonism exhibited by MAb 311.62 and determination of equilibrium dissociation constant for MAb binding and signaling. **A(i).** Competition binding assay of hTSH to TSHR was carried out in presence of NMIgG (134 nM) or in presence of increasing concentrations of MAb 311.62. (**7A(ii)**) Scatchard plots of the binding data shown in Figure **7A** **(i)**. **A(iii).** Plot of the affinity ratios of TSH-TSHR complex as a function of concentration of MAb 311.62. *K_d_’* and *K_d_* are the affinity of TSH to TSH receptor in presence and absence of a given concentration of MAb 311.62 and the curve fitting was done according to the equation mentioned in *inset* (vide text). Cooperativity factor α and the equilibrium dissociation constant for MAb 311.62 derived from the above regression are also mentioned. **B(i).** cAMP dose- response curve to TSH in presence of NMIgG (67 nM), or in presence of increasing concentration of MAb 311.62. *Inset.* Table of *EC_50_* of TSH response to TSHR in presence of increasing concentration of MAb 311.62. **B(ii).** Each shift of hTSH dose response curve as shown in panel **B(i)** yielded a linear regression of equiactive concentrations of hTSH in presence of MAb 311.62. A regression of the respective slopes obtained with the above linear regression is plotted as a function of the concentration of MAb 311.62 according to Eq.2 *(vide text).* Equilibrium efficacy constant (*K_p_*) for MAb 311.62 is 79 nM given by the Y intercept of the linear regression.

**Figure 8:**
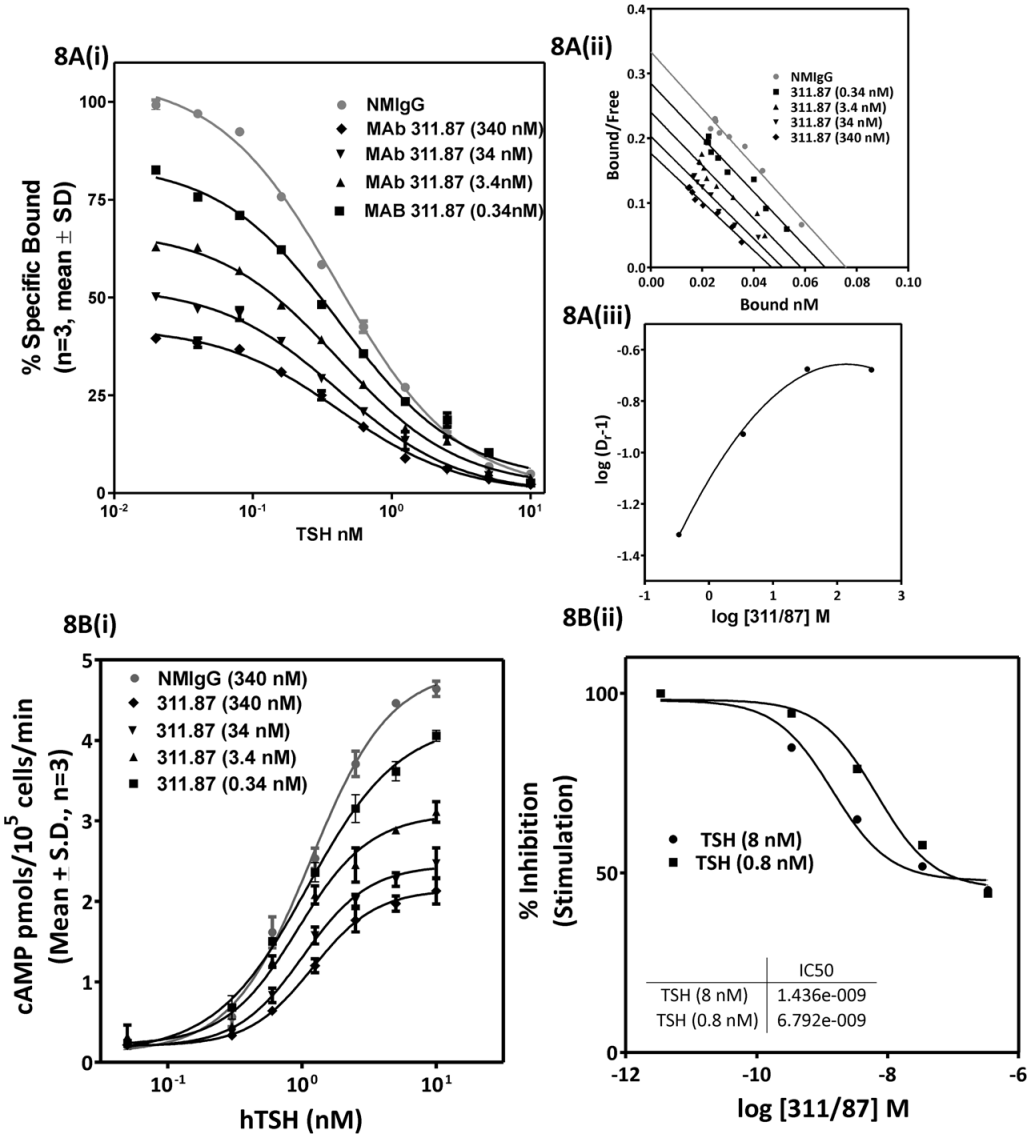
Mechanism of allosteric insurmountable antagonism exhibited by MAb 311.87 and determination of equilibrium dissociation constant of the MAb. **A(i).** Competition binding assays of hTSH to TSHR were carried out in presence of NMIgG (340 nM) or in presence of increasing concentration of MAb 311.87. **A(ii).** Scatchard plot of the same. **A(iii).** Plot of affinity shift (D_r_ versus logarithmic concentration of MAb 311.87 according to the Eq.3. The data points are fit according to a second order polynomial function, where the coefficient of the quadratic term is non-zero (−0.9). **B(i).** cAMP dose response curve to TSH to TSH receptor in presence of NMIgG (340 nM) or in presence of increasing concentration of MAb 311.87. **B(ii).** Effects of MAb 311.87 on responses to 8 nM and 0.8 nM of TSH expressed as a percentage of the control response (No IgG added) plotted as a function of MAb 311.87 concentration to yield an inhibition curve. *Inset* Table of *IC_50_* values for each curve.

Affinity of TSHR was determined in presence of varying concentrations of MAbs 311.62 (Figure 7A(i)) and 311.87 (Figure 8A(i)) keeping the concentration of NMIgG at the highest dose of either antibody. Similarly, dose-response for hTSH was determined in presence of varying concentrations of MAbs (Figure 7B(i) and 8B(i)). Scatchard plots of the binding data showed increasing affinity of the receptor for the hormones in presence of increasing concentration of MAb 311.62 (Figure 7A(ii)), while MAB 311.87 had no such effect (Figure 8A(ii)). To determine whether the increase in affinity in presence of MAb 311.62 is a result of change in receptor conformation, the binding data were fitted into a model of allosteric function as suggested by Kenakin [38] and Price [39]. The cooperatively factor α, which is the measure of affinities in absence and presence of an allosteric modulator was determined by the equation,
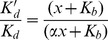
(1)where *K’_d_* and *K_d_* are the dissociation constants in absence and presence of MAb respectively, *x* is the molar concentration of the antibody and *K_b_* is the equilibrium dissociation constant of MAb 311.62-TSH-receptor complex. The graphical fitting of the above equation (Figure 7A(iii)) yielded a value of 2.3 for α and 10 nM for *K_b_* respectively. The α factor value greater than 1 clearly indicates allosteric potentiation of hTSH binding in the presence of MAb 311.62 and this increase in affinity is not related to the orthosteric site of hTSH.

Effect of MAb on TSH stimulated response was contrary to that observed with binding and indicated antagonism by a partial agonist. This partial agonism by the MAb was sufficiently low to allow hTSH to produce further response while shifting the dose response curves with elevated baseline (due to the partial agonism) to the right of the control curve (Figure 7B(i)). The equilibrium dose concentration of MAb 311.62 was calculated using the method of Stephenson modified by Kaumann and Marano [40]. Briefly, for a range of concentrations of MAb 311.62 yielding a range of slopes according to the regressions of equiactive hTSH concentrations, affinity of a partial agonist 311.62 (*K_p_*) can be obtained from the following regression:
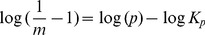
(2)where m is the slope for the regression of equiactive concentrations of hTSH in absence and presence of a particular concentration of partial agonist (*p*) MAb 311.62. The slope of such a regression should be 1, and the experimental slope was found to be 1.03. The value of affinity constant of MAb 311.62 so obtained was found to be 79 nM (Figure 7B(ii). The difference in *K_b_* and *K_p_* for MAb 311.62 and the amplification ratio (*K_d_*/*EC_50_*) <1 indicate decoupling of the binding of hTSH from that of receptor stimulation through allosteric transition [41].

In case of MAb 311.87, the above analysis could not be performed because of the insurmountable antagonism exhibited by this antibody for hTSH binding. In order to determine whether this antagonism was due to orthosteric or allosteric interactions, a modified method of Christopoulos and Kenakin [42] was used. The method depends on the deviation from the linearity of unitary slope in the regression,
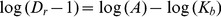
(3)where (D_r_) is the affinity shift, A is the ratio of hTSH affinity in presence and absence of each concentration of MAb and *K_b_* is the apparent equilibrium constants for the antibody. As shown in Figure 8A(iii), the regression deviates significantly from linearity suggesting allosteric insurmountable antagonism. *K_b_* for MAb 311.87 could not be determined satisfactorily because of the non-linearity of the above regression and hence, TSH binding in presence of MAb 311.87 was fit according to the equation proposed by Ehlert [43].
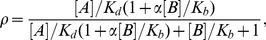
(4)Where 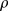 is the specific binding of TSH to TSHR, 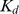 and 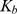 are the association constants for hTSH and the MAb respectively, and the concentrations of hormones and MAb are denoted by [A] and [B] respectively. The value of the cooperativity factor α, described previously, was <1 (0.337) and 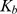 was 1.5 nM suggesting that MAb 311.87 is a negative allosteric modulator of hormone binding.

MAb 311.87 inhibition curves (Figure 8B (ii)) derived from TSH dose response curves in presence of increasing concentrations of the MAb (Figure 8B(i)) yielded *IC_50_* values of 1.43 nM for 8 nM of TSH and a value of 6.9 nM for 0.8 nM TSH. It can be seen that inhibition curve shifts to the left with increasing concentrations of TSH, indicating an allosteric mechanism whereby MAb 311.87 blocks signaling without changing the affinity of the receptor for the hormone.

Ability of the MAb 311.62 to increase the affinity of the receptor to the hormone may also be argued to be caused by oligomerization of the receptor, a predominant notion to explain negative cooperativity in TSHR. The intrinsic bivalent nature of the antibody would also support the above argument. However such an increase in the affinity would undoubtedly be associated with increase in efficacy, a scenario not encountered in case of MAb 311.62. Moreover, avidity of such bivalent antibody has been found to be higher for the cell surface antigen than its corresponding F_AB_, as seen in the cases of antibodies against HLA-A2 [44]. However, this is not the case with MAb 311.62 as radiolabelled IgG of MAb 311.62 displayed equivalent or slightly lower affinity than the F_Ab_ fragments (Figure S9). Moreover, the B*_max_* displayed by both the antibody forms were comparable suggesting TSHR-MAb 311.62 interaction is predominantly monovalent. Although the present data suggest that the mechanism of action of MAb 311.62 does not involve receptor dimerization, this does not preclude the possibility of the association of receptor dimerization and negative cooperativity, inherent to TSHR.

### Conformational Change in MAb 311.62 Epitope following Hormone Binding: Probable Interaction of the Hormone α-subunit with the TSHR Hinge Region

**Figure 9:**
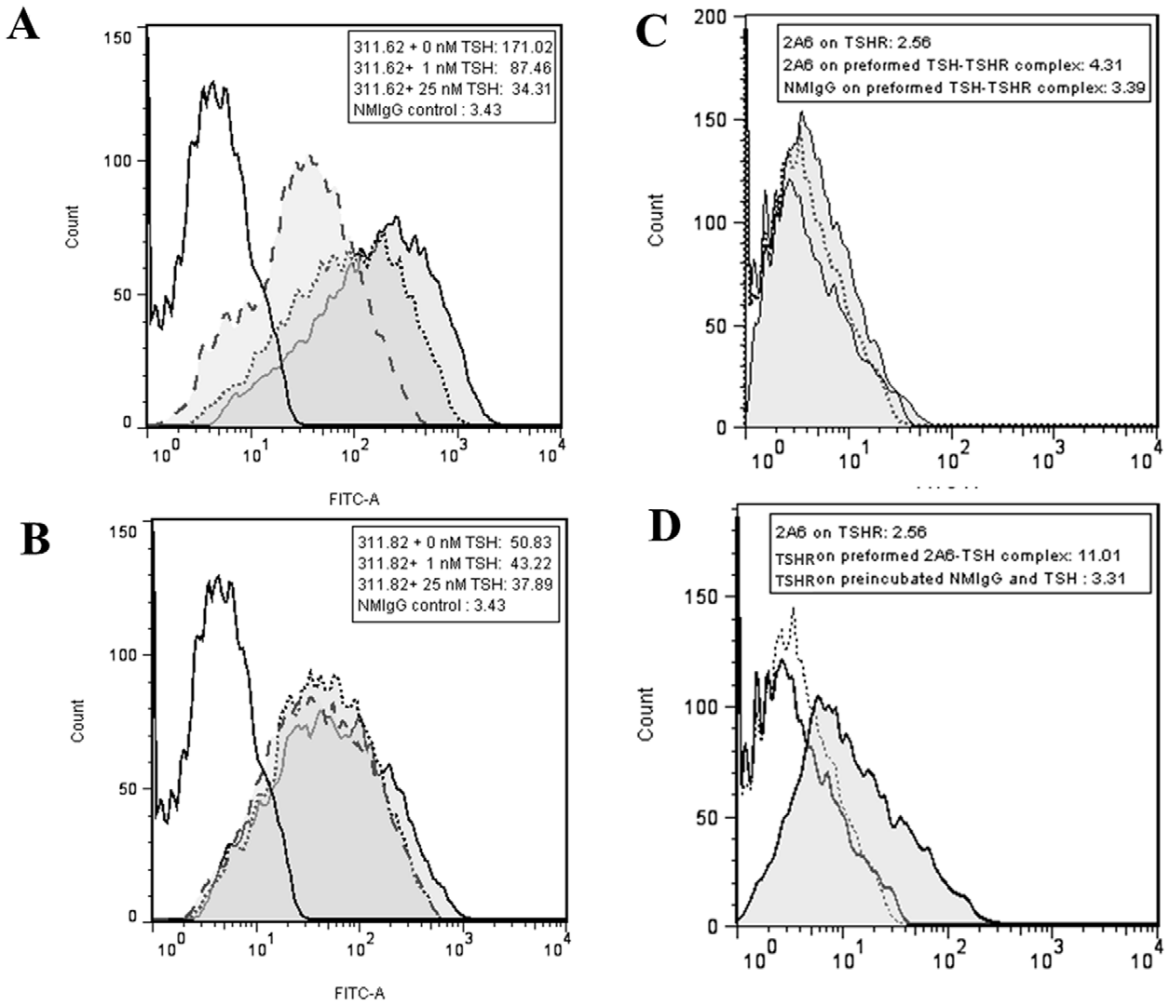
Involvement of TSHR-hinge region and hormone α-subunit in hormone mediated conformational change in TSHR. Flow cytometric analysis of binding of 25 µg/ml of (**9A.**) MAb 311.62 or (**9B.**) MAb 311.82 *to* HEK293-hTSHR cells in absence of TSH (shaded solid line histogram) or pre-incubated with either 1 nM TSH (shaded dotted line histogram) or 25 nM TSH (shaded dashed line histogram). The culture supernatant of hormone α-subunit specific monoclonal antibody 2A6 was either added to HEK293-hTSHR cells previously incubated with (**9C.**) 25 nM TSH or (**9D.**) 25 nM TSH was added to HEK293-hTSHR cells previously exposed to similar concentration of 2A6 and binding of 2A6 to TSH in a TSH-TSHR complex was monitored in flow cytometry. All the flow cytometric experiments were carried out at 4°C to prevent hormone mediated receptor internalization.

Conformational change upon hormone binding has been previously reported for FSH receptor [45] by determining the change in CD spectra of isolated FSHR ECD in presence of FSH. A more elaborate procedure described for CCR5 [46] and µ-opoid [47] receptors was the use of MAbs to detect ligand induced conformational change in the receptor. Conformational changes in TSHR post- TSH binding was investigated by determining the binding of MAb 311.62 to the receptor after preincubating the TSHR cells with the hormone in flow cytometric analysis. As shown in Figure 9A, there was a concentration (of hormone) dependent decrease in binding of MAb 311.62 while that of the neutral MAb 311.82 remained unaffected (Figure 9B) clearly indicating that loss of MAb 311.62 binding is consequent to the specific ligand mediated conformational change at the hinge region.

Conversely, accessibility of the hormone subunits in a TSHR-311.62 complex was monitored using the hormone α-subunit specific MAb, 2A6. This MAb binds to both ^125^I-hCG and ^125^I-hTSH (Figure S7A), inhibits hCG binding to LHR while exhibiting no effect on TSH –TSHR interaction (Figure S7B). The MAb, by itself, does not bind to HEK 293-TSHR cells (Figure 9C), but the complex of this MAb with the hormone could still bind to the HEK293-hTSHR suggesting its epitope in the hormone α-subunit is not involved in the primary interaction of TSH and TSHR. However, the antibody failed to bind to hormone when it is previously complexed with the receptor indicating loss of epitope post hormone binding (Figure 9D). Loss of binding of either MAb in a preformed hormone-receptor complex may be explained by either change in conformation of its epitope or by inaccessibility arising from such a change.

## Discussion

Extensive mutational analysis of the hinge region residues has shown the importance of the hinge region in hormone binding and receptor stimulation [7]. These mutations, while providing valuable insights into receptor functioning, suffer from the stabilization of a Hammond state in the structural segment containing the mutation [48]. By increasing or decreasing the energy barrier of activation, the mutation could allosterically stabilize a receptor conformation unsuitable for hormone binding, which may be misconstrued to be a part of the hormone-binding site. Moreover, substitution of residues at the hinge region and LRR domain junction by charged residues cause misfolding of TSHR causing impairment of cell surface expression [49]. MAbs on the other hand have the unique capability of differentiating between an allosteric and an orthosteric receptor site and have proved to be reliable tools in detecting the ligand induced conformational changes in the wild type or mutated receptors. TSHR stimulatory monoclonal antibodies have been instrumental in understanding TSH interaction with the LRR domain of TSHR [50] or the N terminal region of ECD in receptor activation [51]. Almost all the reported TSH stimulatory MAbs had epitopes in the LRR domain except TSAB-4 [52] that recognized in the TSHR hinge region [53] or all were full agonists except IRI-SAb1 [54] and affected TSH binding. In contrast, MAb 311.62 is a hinge region specific partial agonistic MAb that does not inhibit TSH binding but stimulates the receptor. In the present study, MAB 311.62 and the antagonistic MAB 311.87 have been used to understand the mechanisms of hormone binding and receptor activation.

### Initiation of Hormone Induced Conformational Change may Originate at LRR 7–9

It is expected that transmission of signal from the LRR to the TMD would require considerable conformational change in the receptor. It is highly unlikely that contact of the hormone at LRR 4–6 alone can initiate such a conformational change owing to its rigid structure and hence requires envisioning of an extended hormone binding site. In the present study, the antagonistic MAb 311.87, recognizing LRR 7–9 (aa 201–259) provides insights into the conformational change initiated by hormone binding.

The crystal structure of FSH-FSHRECD indicates that residues in LRR 7–9 region are in contact with the β-loop2 of the hormone. While the LRR 7–9 region seems to be important for hormone binding and signaling as indicated by mutational studies [55], the β loop2 appears to be of important for subunit stabilization alone and not for hormone specificity [56]. The role of LRR 7–9, at least in case of TSH receptor, becomes apparent by studying the allosteric insurmountable antagonism of MAb 311.87. This MAb binds to a site distal from the orthosteric site, alters the accessibility of the hormone to the hormone-binding domain and reduces its affinity for the hormone, thus indicating that the possible interaction between β-loop2 and LRR 7–9 initiates hormone induced conformational changes leading to receptor activation. The allosteric nature of the antibody is also indicated by the observation that inhibition of response to the hormone by the antibody is more pronounced at the higher concentration of the hormone with the leftward shift of inhibition curves (Figure 8B (ii)). This change in the conformation is independent of the hinge region as indicated by inhibition of hTSH binding to TSHR/LHR chimeras (Table 5) and seems specific to this region alone as the two MAbs 413.1.F7 and 311.82 recognizing the extreme N terminal and C- terminal domains of the ECD respectively had no effect on hormone-receptor interactions. In addition, the similarity in the values of the equilibrium binding constant of MAb 311.87 (*K_b_*) and *IC_50_*, suggests that this region is not involved in coupling of hormone binding to response. Thus, the role of TLRR 7–9 in receptor activation is primarily to provide a conformational flexibility during or after hormone binding without being directly involved in the primary interactions between the hormone and the receptor. The presence of broken β loop β motif in the C-terminal region of the LRR domain has been thought to comprise of the 11^th^ LRR in the ECD [49] and may contribute to the initiation of TSH induced conformational change, hence acting as the “neck or pivot” of the hinge region in the ECD.

### MAb 311.62 Binds to “Open” Receptor State

The epitope recognized by MAb 311.62 was located in the aa 265–275 in the N-terminal region of the cysteine box 2 adjacent to the highly conserved “YPSHCCAF” motif. Mutations such as S281I and C284S [26] in this motif render the receptor constitutively active, indicating the residues in this stretch are involved in transition from the “closed” low basal state to an “open” high basal state of the receptor [8]. Close packing between the Cb-2 residues and ECL1/2 have been implicated for maintaining the receptor in the low basal state [30]. Mutations at the conserved serine (S281), ECL1 (I486F) or ECL2 (I568T) disrupt this environment giving rise to an open high basal receptor state. Increase in binding of MAb 311.62 to I486F or I568T suggests the preferential binding of MAb 311.62 to such an open receptor state thus stabilizing the high basal conformation. However, MAb cannot further stimulate these mutants, probably due to a conformational lock in the hinge region. This may also explain the non-responsiveness of these mutants to the hormonal stimulation. The fact the MAb does not bind to E251K or the receptor complexed with the hormone further confirms the conformational nature of the epitope.

### Coupling of Hormone Binding and Receptor Activation Occurs at Cysteine Box-2

The effect of MAb 311.62 on wild type TSHR bears similarity to the mutations in the Cb-2 region such as increase in the basal cAMP production, increase in affinity for TSH and decrease in the responsiveness to the hormone. Dextral shift in hormone dose-response curves in presence of increasing MAb concentration with concomitant progressive increase in the hormone affinity indicates uncoupling between hormone binding and activation of the receptor caused by the antibody. This would also indicate that hormone binding and receptor activation are distinct events where the antibody facilitates hormone binding, but prevents the occurrence of the second step of receptor activation. Mechanistic studies of the partial agonism displayed by MAb 311.62 show a positive allosterism for binding and a negative allosterism for receptor activation, with a signaling ratio (*K_B_/K_P_*) of <1 further validating this hypothesis [38]. The actual event of signal transmission from the LRR may be conveyed to the TMD through the contact of the α-subunit of the hormone to the hinge region as suggested by the loss of epitope of the α subunit specific MAb 2A6 in a preformed hormone-receptor complex. This observation is in agreement with the model of receptor activation proposed by Fan and Hendrickson [1] and also with the observation that an hormone α-subunit specific MAb was capable of binding to hCG complexed with first 297 residues of LHR, but not to hCG complexed with intact receptors [57]. Although interaction between the hormone α-subunit and the hinge region of TSHR has been demonstrated using bTSH mutants or the superagonist TSH analog TR1401 [58], direct evidence of contact between the αL1 and αL3 of TSH to the receptor ECLs has not been forthcoming.

### The Inverse Agonistic Activity of the Hinge Region is Independent of the LRR

Our earlier studies showed that deletion of the ECD or aa 296–331 in the hinge region of FSHR increased the basal cAMP production [13]. A similar increase in the basal activity was found for TSHR when the entire ECD was deleted. In both cases, presence of TSHR or FSHR hinge region suppressed this high basal activity These, as well as, the previously reported data suggest that the hinge regions of TSHR and FSHR act as tethered inverse agonist [59] and dampen the constitutive activities of the TMD. Ability of MAb 311.62 to stimulate LRR deleted TSH receptor, similar to that observed in case of FSHR by its stimulatory antibody [13], also confirms the tethered inverse agonism of the hinge region of these receptors and perhaps the contact of the hormone α-subunit (as suggested by MAb 2A6) or by the TSH superagonist TR1401 [58] with the hinge region leads to a conformational change and ultimately receptor activation.

The mechanism of TSHR has been extensively investigated, particularly with the help of the stimulatory autoantibodies in the Graves’ thyrotoxicosis patients. Stimulatory monoclonal antibodies, both mouse and human, have been generated to gain insight into the action of these autoantibodies as well as to gain an overall insight into the receptor activation process. As mentioned above, reports of TSHR agonistic antibodies with epitopes in the hinge region have been rare with the sole exception of TSAB-4 which recognized full length TSHR but not the hinge region chimeric construct TSH-LHR-6 [53]. Most of the agonistic MAbs, such as M22, while providing insights into the commonalities between binding of autoantibodies and hormone to LRRD, provide little insights into the role of the hinge region in the receptor activation. This is mainly due to the fact that epitopes of these antibodies overlap with LRRD and cannot be used to delineate the role of hinge in hormone binding and receptor activation. The fact that several TSHR HinR specific MAbs such as 2C11 (aa 252–262) [60], A7 (402–415) [61], and TAB-6 (aa 316–335) [62] do not compete with TSH for receptor bindig except for the MAbs recognizing the region 381–385 [63] suggests indirect involvement of the hinge region in hormone binding. This is similar to that observed with MAb 311.62 which has does not inhibit hormone binding, but increases the affinity of the receptor for the hormone. On the other hand, similarity may be drawn between MAb 311.87 and antibodies such as TAB-8 or 10A11 that bind to “epitope B” in the α-subunit of TSHR ECD [64]. According to Davies et.al, these antibodies may bind in part to the LRR region but do not bring about the required structural change for signal transduction. yet are still able to hinder TSH binding to this site [65]. In this study we propose that hindrance in TSH binding by such antibodies may not be through direct competition, but due to allosteric inhibition of hormone binding. This would suggest that while the LRR 7–9 region may be important for conveyance of signal generated by hormone-receptor interaction, the Cb-2 region is important for both hormone binding and receptor activation, more precisely the coupling of hormone binding and receptor activation. Based on the mechanistic insight gained from the action of MAb 311.62 on TSHR, we proposed that this coupling supports a conformational selection model of receptor activation where the hormone binds preferentially to an “open” state receptor characterized by loss of constraint of the hinge region-TMD interactions and a concomitant increase in the basal activity as compared to a “closed” hinge constrained receptor state. As a corollary, we also propose that the conformational state of the hinge is bidirectional that is while the binding of hormone to LRRD causes reorganization of the hinge region subdomains, the hinge region itself might affect the LRRD orientation and makes this domain more or less accessible to the hormone. In this regard, Rapoport and co-workers, based on the effects of TSH and MAbs CS-17 and M22 [2] on mutation in the interfacial residues of LRRD and hormone, have suggested that the LRRD is tilted forward in its interface with the hinge region [66]. The mechanistic analysis of the antagonistic MAb 311.87, in addition to providing credence to this possibility, also suggests that, at least in case of TSH, the β-subunit Loop2 is responsible for disengagement of this interface causing further rearrangements in the hinge region.

To summarize, we suggest that the hinge region acts as a bimodal switch capable of fine-tuning both hormone binding and response and describes the hormone binding as a function of the basal state of the receptor, and that a direct correlation may exist between conformational changes in the hinge region and the affinity of the receptor for the hormone.

## Supporting Information

Figure S1 **A**. **Schematic representation of different overlapping regions** of TSH receptor exodomain comprising of the first three leucine rich repeats. **S1B(i)** TLRR 1–3; **S1B(ii)** TLRR 4–6; **S1B(iii)** TLRR 1–6; and **S1B(iv)** TLRR 7-HinR; were expressed as GST fusion protein in E.coli strain BL21 and purified through GST affinity chromatography. The purity of the receptor fragments were verified by immunoblot analysis using GST antisera where no cross reactivity was observed in the vector only transformed cells **S1B(iv).** The hinge region of TSH receptor (**S1B(v))** TSHR HinR; was expressed with an N-6x-His tag and purified using Ni^+^2-NTA IMAC chromatography and purity was verified by immunoblot analysis using Anti-His tag monoclonal antibody. TSHR ECD was purified form the supernatant of pichia pastoris expression system using phenyl-Sepharose hydrophobic interaction chromatography (HIC) followed by Sephacryl S200 size exclusion chromatography and purity ascertained by immunoblotting with TSHR polyclonal antibody against TLRR 1–6 (**S1B(vi)**).
(TIF)

Figure S2 **Characterization of HEK293 cell line expressing hTSHR.** Stable cell line expressing hTSHR was treated with increasing concentrations of hFSH/bTSH for 15 min at 37°C in a 5% CO2 atmosphere, and the total cAMP produced was determined by RIA *(vide text)*. *Inset*, Scatchard plot of the binding data carried out with the membrane preparation. Results presented are representative of several independent experiments.
(TIF)

Figure S3 **Immunological characterization of TSHR polyclonal antibodies. S3A.** Various fragments of TSHR (50 ng/well) or GST were adsorbed on to a plastic surface and incubated with 100 µl of each dilution of either antibodies raised against TLRR 1–6 fragment **(S3A(i))** or against TSH HinR fragment **(S3A(ii))** followed by addition of goat anti-rabbit IgG-peroxidase and determination the enzyme activity. GST specific antibodies in TLRR 1–6 was removed by negative affinity chromatography. **S3B.** The HEK293-TSHR or HEK293 cells were fixed with 4% paraformaldehyde and incubated with 10 µg/ml of either **(S3B(i))** TLRR 1–6 IgG or **(S3B(ii))** TSHR HinR IgG for 1 h at 37°C followed by incubation with FITC-conjugated anti-rabbit antibody (1∶500) for an additional 45 min. Confocal images were obtained were obtained with a Leica SP5-AOBS confocal laser scanning microscope. The HEK 293 cells and HEK293-TSHR cells treated with NR-IgG were used as controls (Not shown). Each picture is a representative of at least two independent experiments.
(TIF)

Figure S4 **Characterization of TSHR Monoclonal antibodies: Flowcytometric analysis.** HEK293-TSHR (shaded) or HEK 293 cells (unshaded) were washed with PBS after detaching the cells with Ca^+2^/Mg^+2^ free PBS and incubated with 25 µg/ml dilutions of different TSH receptor MAbs in PBS containing 5% FBS at 4°C for 1 h. Cells were then washed twice and incubated at 4°C for 1 h with a 1∶500 dilution of FITC-conjugated secondary antibody (Sigma). The excess secondary antibody was washed with PBS and the cells in a FACSCANTO II (Becton-Dickinson, Franklin Lakes, NJ, USA), flowcytometer. The HEK 293 cells and HEK293-TSHR cells treated with NRIgG were used as controls. Each picture is a representative of several independent experiments.
(TIF)

Figure S5 **Characterization of TSHR Monoclonal antibodies: Immunocytochemistry.** The HEK293-TSHR cells were fixed with 4% paraformaldehyde and incubated with 2.5 µg/ml of different TSH receptor monoclonal antibody or with NMIgG for 2 h at 37°C followed by incubation with FITC-conjugated anti-rabbit antibody (1∶500) for an additional 45 min. Confocal images were obtained were obtained with a Leica SP5-AOBS confocal laser scanning microscope. 10 µg/ml of IgG against TLRR 1–6 was used as a positive control.
(TIF)

Figure S6 **Cross-Reactivity of MAb 311.62 with hFSHR and hLHR.** HEK293 cells expressing hTSHR, hLHR or hFSHR are mock transfected were incubated with 10 µg/ml of MAb 311.62 and processed for flowcytometric analysis as mentioned in supplementary Figure 4. Histograms were created for 10000 events for each and Median Fluorescence Intensity (MFI) was calculated for each. The shaded histogram corresponded to MAB 311.62 binding to HEK293-TSHR where as non-shaded histogram with solid line corresponded to LHR, broken line to that of FSHR and grey line to that of mock transfected HEK 293 cells.
(TIF)

Figure S7 **Characterization of α-subunit specific monoclonal antibody 2A6. S7A. Binding of 2A6 to labelled hCG/hTSH.** 0.1–0.2 µCi (∼100,000 cpm) of 125I-hCG/125I-hTSH were incubated overnight with increasing dilutions of 2A6 antibody at room temperature (28–30°C). The antigen–antibody complexes were precipitated by centrifugal separation at 4000 g after the addition of goat- anti-mouse IgG and PEG-6000.The supernatant was discarded and the radioactivity in the pellet was counted using Perkin-Elmer auto gamma counter
**S7B. Effect of 2A6 on binding hCG/hTSH to their cognate receptor** Effect of 2A6 on hormone–receptor interaction was determined by incubating the increasing dilution of 2A6 with either 125I-hCG or 125I-hTSH for 1 h at room temperature followed by addition of membrane preparation of the HEK293-LHR or HEK293-TSHR respectively and continuing the incubation for additional 1 h. labelled-hormone bound to the receptor was pelleted by centrifugation at 4000×*g* and counted in a gamma counter. Specific binding was obtained by subtracting the bound counts from non-specific binding obtained by addition of 0.5 µg of unlabelled hormone to each reaction mixture. Percentage ratio between difference of counts in presence and absence of 2A6 to the specific binding denoted the % inhibition of receptor binding by 2A6.
(TIF)

Figure S8 **Stimulation of HEk292-hTSHR cells by FAb prepared from MAb 311.62 IgG.** HEK293-hTSHR cells were incubated with increasing concentration of MAb 311.62 IgG or FAb fragments prepared from the in the absence of hTSH for 1 h at 37°C, and cAMP produced was determined by RIA. The solid and the dotted lines represent the cAMP produced by HEK293-hTSHR in presence of saturating concentrations (1 µM) of NMIgG or FAb prepared from NMIgG.
(TIF)

Figure S9 **Receptor binding of radioiodinated FAB and MAb.** HEK293-TSHR membrane preparation (10 µg) was incubated with different concentrations of FAb/Mab 311.62 in presence of 10 nM of ^125^I-FAb/MAb (specific activity tracer - 0.10 µci/fmol) at 37°C for 2 h in a reaction volume of 250 µl. The receptor bound radioactivity was centrifugally separated (4000 g at 4°C for 20 minutes) after addition of 2.5% PEG 6000 and counted in Perkin Elmer γ-counter. The non-specific binding was determined in presence of excess unlabeled antibody(10 µg/ml).
(TIF)

Figure S10 **Scatchard Analysis of TSH-TSHR binding at excess TSH concentrations in** **presence of antibodies.** ^125^I-hTSH (20000 CPM) was incubated with hTSHR membranes (20 µg/ml) with increasing concentrations (upto 1 µg/ml) of the unlabelled hTSH in the absence or presence of 50 µg/ml of A. 311.82 IgG, B. 311.87 IgG, C. 311.174 IgG or D. 311.62 IgG and the binding data converted into Scatchard plots. Two linear regression was resolved from the curvilinear Scatchard plot as described by De meyts *etal,* 1975. Solid lines denote the high affinity receptor component (apparent affinity, K_d1_ = 0.11 nM in presence of NMIgG) whereas the broken lines represent low affinity receptor component (apparent affinity, K_d2_ = 9.8 nM in presence of NMIgG)
(TIF)

Table S1 **Isotyping of TSHR MAb.** 1 µg/ml of IgG from subclones of each TSHR MAb was coated on polystyrene plates (Nunc Immunsorb) and indirect ELISA was carried out using mouse isotype specific antibodies (Mouse Monoclonal Antibody Isotyping Reagents, Sigma-Aldrich) as per the manufacturer’s specification. The Light chain subtype was determined by a mouse monoclonal antibody isotyping kit (dipstick format, GIBCO, BRL).
(DOC)
