# Supplementary figures and images for: The Hinge Region of Human Thyroid-Stimulating Hormone (TSH) Receptor Operates as a Tunable Switch between Hormone Binding and Receptor Activation
Source: PLoS One. 2012 Jul 6;7(7):e40291. doi: 10.1371/journal.pone.0040291 (PMC3391290; doi:10.1371/journal.pone.0040291)

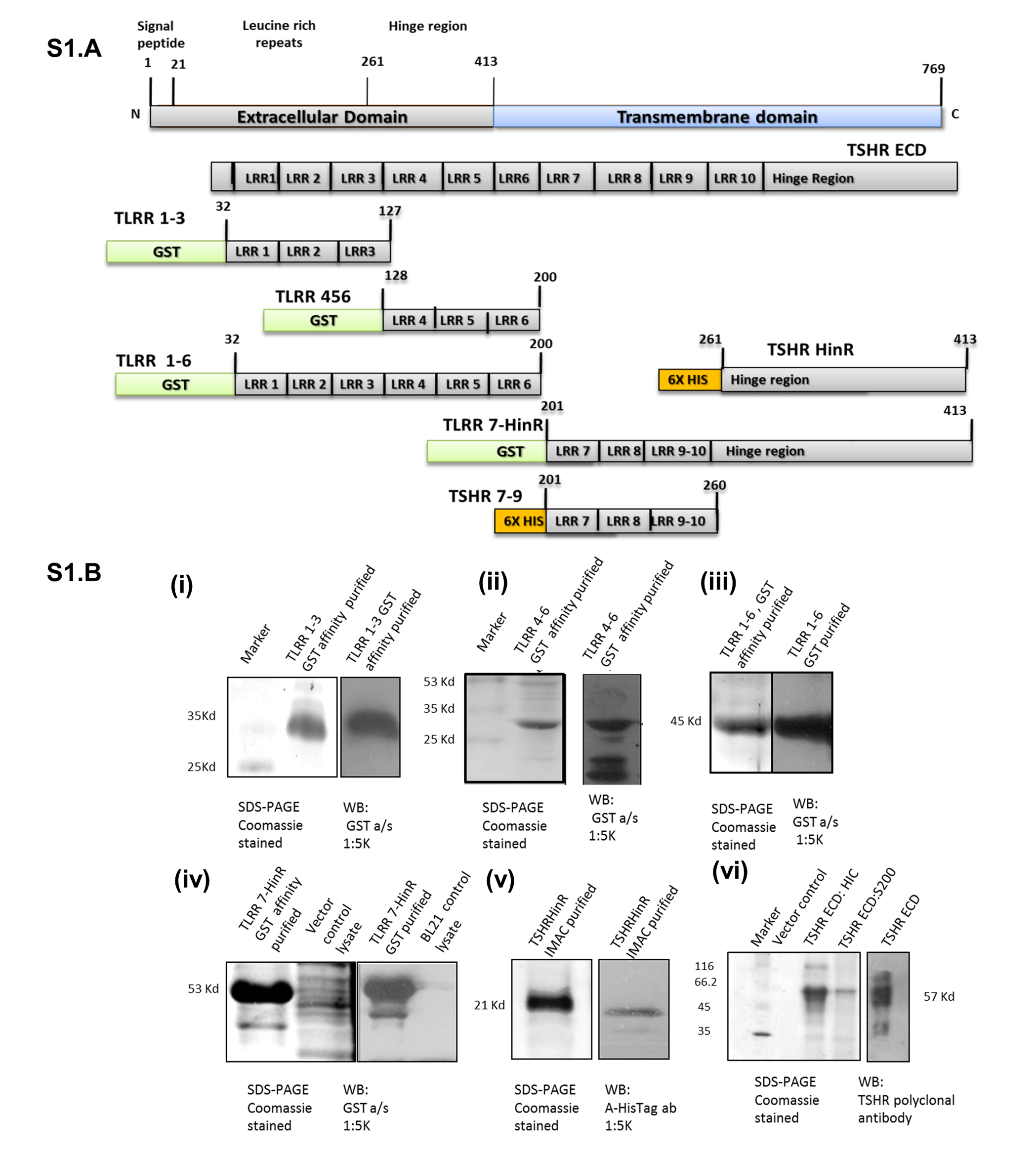

Supplement: Figure S1 — A. Schematic representation of different overlapping regions of TSH receptor exodomain comprising of the first three leucine rich repeats. S1B(i) TLRR 1–3; S1B(ii) TLRR 4–6; S1B(iii) TLRR 1–6; and S1B(iv) TLRR 7-HinR; were expressed as GST fusion protein in E.coli strain BL21 and purified through GST affinity chromatography. The purity of the receptor fragments were verified by immunoblot analysis using GST antisera where no cross reactivity was observed in the vector only transformed cells S1B(iv). The hinge region of TSH receptor ( S1B(v)) TSHR HinR; was expressed with an N-6x-His tag and purified using Ni+2-NTA IMAC chromatography and purity was verified by immunoblot analysis using Anti-His tag monoclonal antibody. TSHR ECD was purified form the supernatant of pichia pastoris expression system using phenyl-Sepharose hydrophobic interaction chromatography (HIC) followed by Sephacryl S200 size exclusion chromatography and purity ascertained by immunoblotting with TSHR polyclonal antibody against TLRR 1–6 ( S1B(vi) ). (TIF) [file pone.0040291.s001.tif]

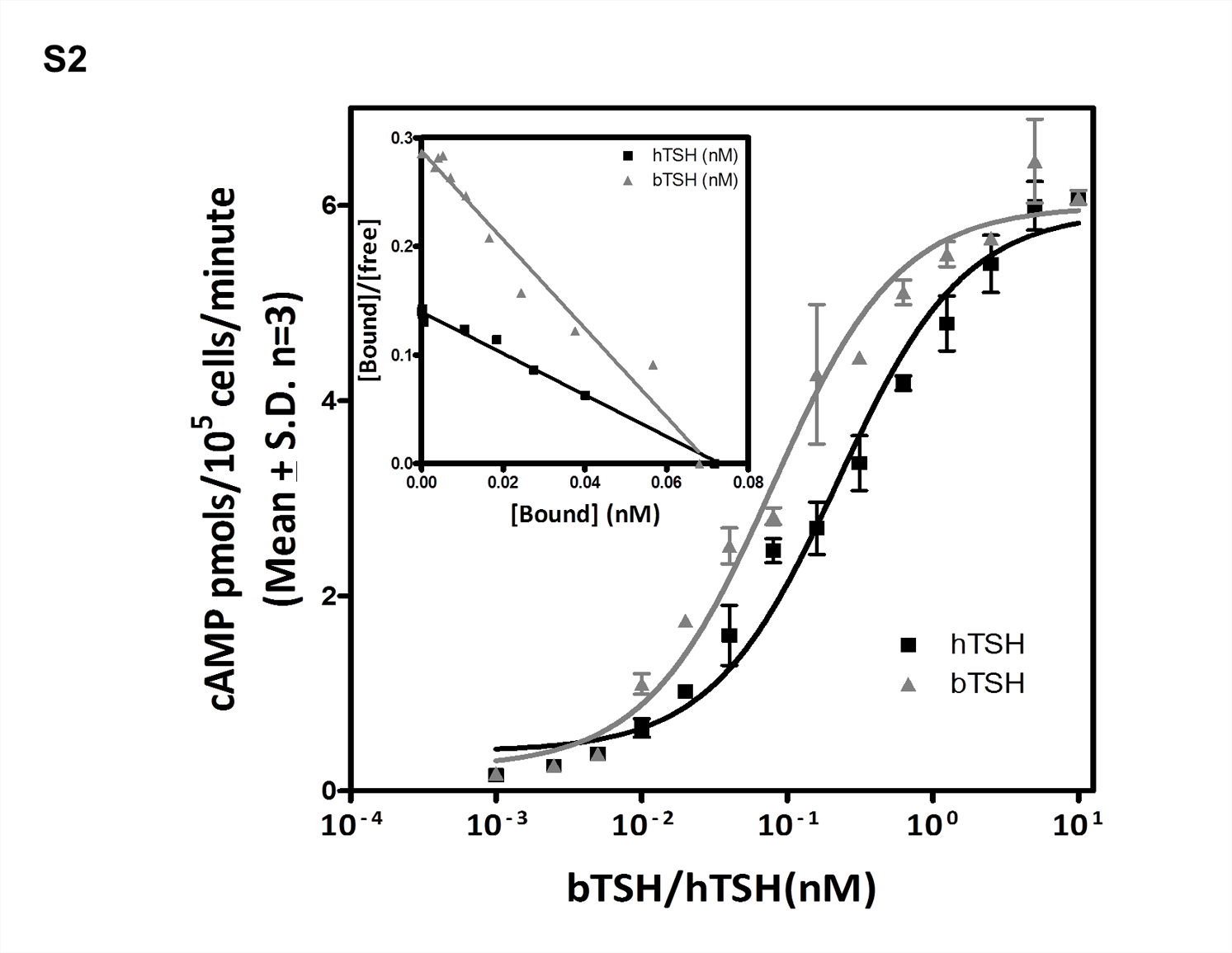

Supplement: Figure S2 — Characterization of HEK293 cell line expressing hTSHR. Stable cell line expressing hTSHR was treated with increasing concentrations of hFSH/bTSH for 15 min at 37°C in a 5% CO2 atmosphere, and the total cAMP produced was determined by RIA (vide text). Inset, Scatchard plot of the binding data carried out with the membrane preparation. Results presented are representative of several independent experiments. (TIF) [file pone.0040291.s002.tif]

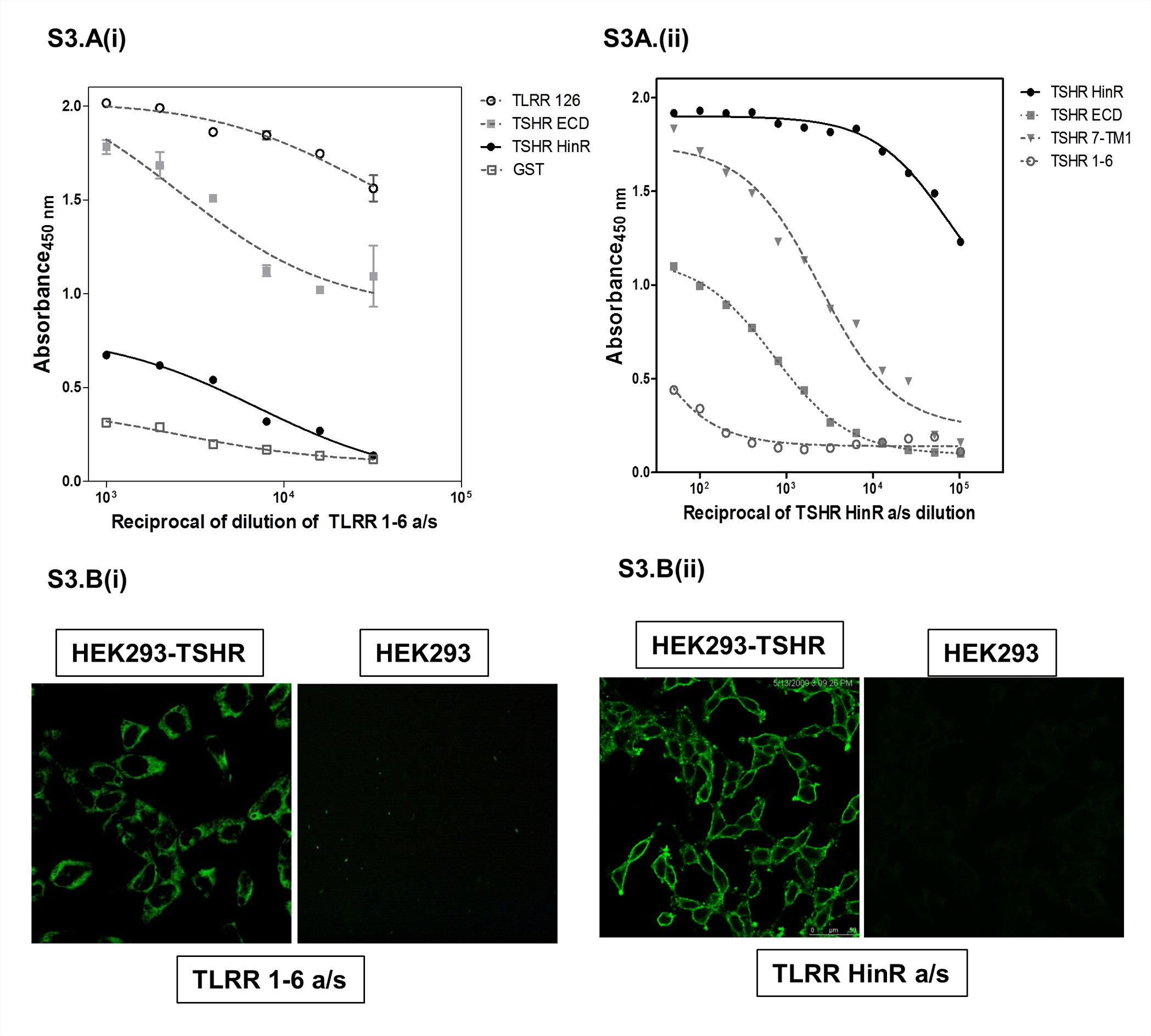

Supplement: Figure S3 — Immunological characterization of TSHR polyclonal antibodies. S3A. Various fragments of TSHR (50 ng/well) or GST were adsorbed on to a plastic surface and incubated with 100 µl of each dilution of either antibodies raised against TLRR 1–6 fragment (S3A(i)) or against TSH HinR fragment (S3A(ii)) followed by addition of goat anti-rabbit IgG-peroxidase and determination the enzyme activity. GST specific antibodies in TLRR 1–6 was removed by negative affinity chromatography. S3B. The HEK293-TSHR or HEK293 cells were fixed with 4% paraformaldehyde and incubated with 10 µg/ml of either (S3B(i)) TLRR 1–6 IgG or (S3B(ii)) TSHR HinR IgG for 1 h at 37°C followed by incubation with FITC-conjugated anti-rabbit antibody (1∶500) for an additional 45 min. Confocal images were obtained were obtained with a Leica SP5-AOBS confocal laser scanning microscope. The HEK 293 cells and HEK293-TSHR cells treated with NR-IgG were used as controls (Not shown). Each picture is a representative of at least two independent experiments. (TIF) [file pone.0040291.s003.tif]

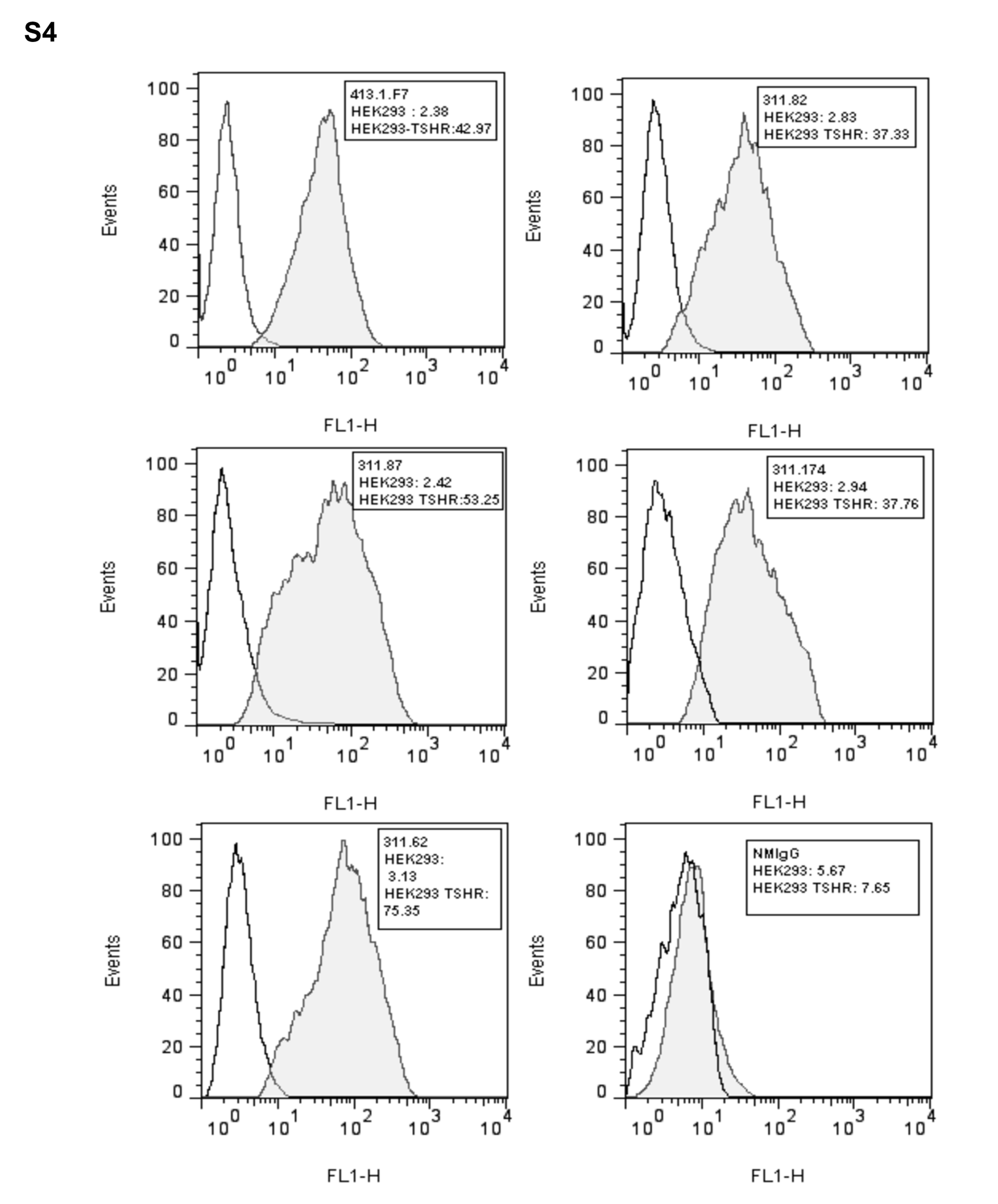

Supplement: Figure S4 — Characterization of TSHR Monoclonal antibodies: Flowcytometric analysis. HEK293-TSHR (shaded) or HEK 293 cells (unshaded) were washed with PBS after detaching the cells with Ca+2/Mg+2 free PBS and incubated with 25 µg/ml dilutions of different TSH receptor MAbs in PBS containing 5% FBS at 4°C for 1 h. Cells were then washed twice and incubated at 4°C for 1 h with a 1∶500 dilution of FITC-conjugated secondary antibody (Sigma). The excess secondary antibody was washed with PBS and the cells in a FACSCANTO II (Becton-Dickinson, Franklin Lakes, NJ, USA), flowcytometer. The HEK 293 cells and HEK293-TSHR cells treated with NRIgG were used as controls. Each picture is a representative of several independent experiments. (TIF) [file pone.0040291.s004.tif]

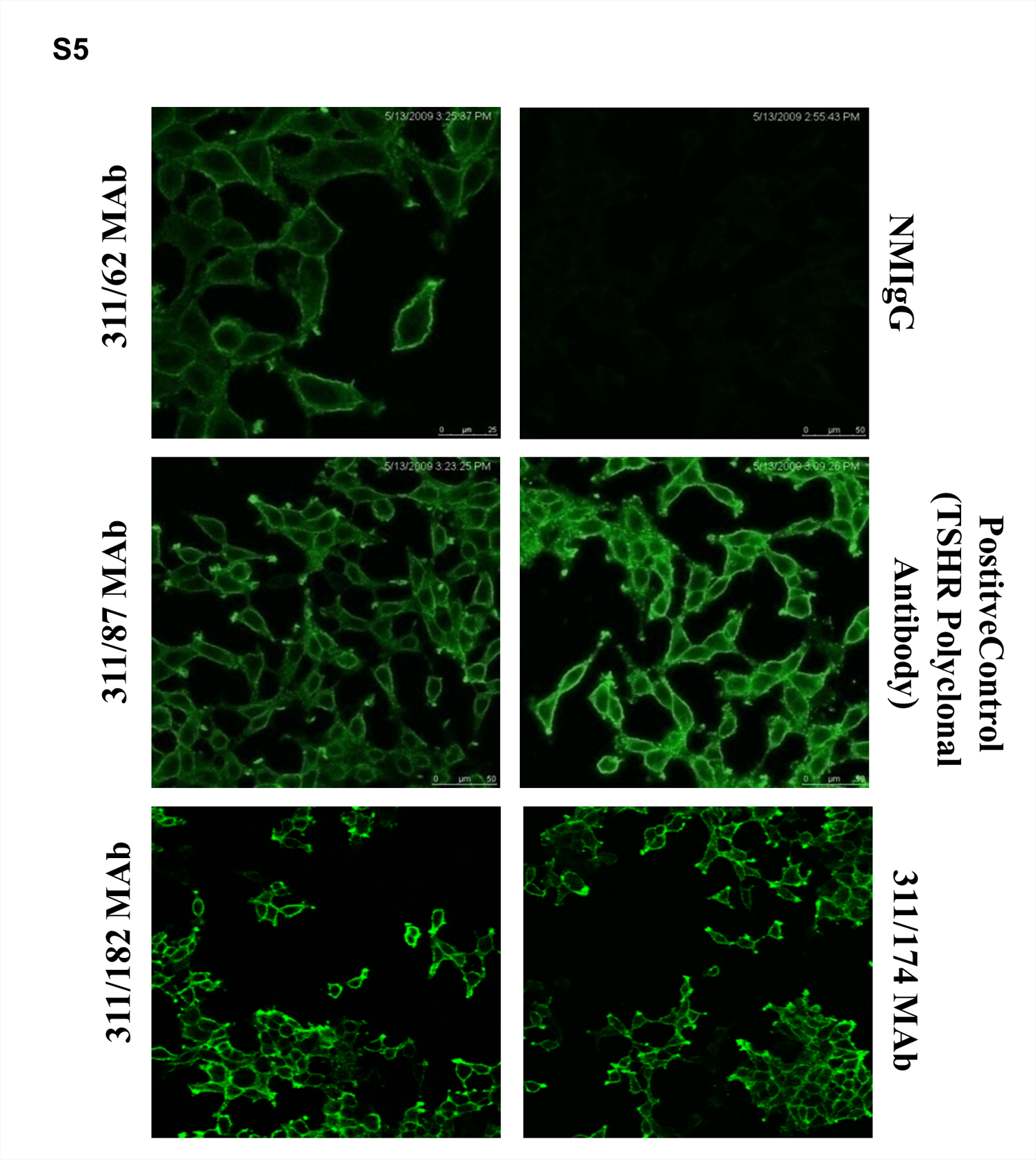

Supplement: Figure S5 — Characterization of TSHR Monoclonal antibodies: Immunocytochemistry. The HEK293-TSHR cells were fixed with 4% paraformaldehyde and incubated with 2.5 µg/ml of different TSH receptor monoclonal antibody or with NMIgG for 2 h at 37°C followed by incubation with FITC-conjugated anti-rabbit antibody (1∶500) for an additional 45 min. Confocal images were obtained were obtained with a Leica SP5-AOBS confocal laser scanning microscope. 10 µg/ml of IgG against TLRR 1–6 was used as a positive control. (TIF) [file pone.0040291.s005.tif]

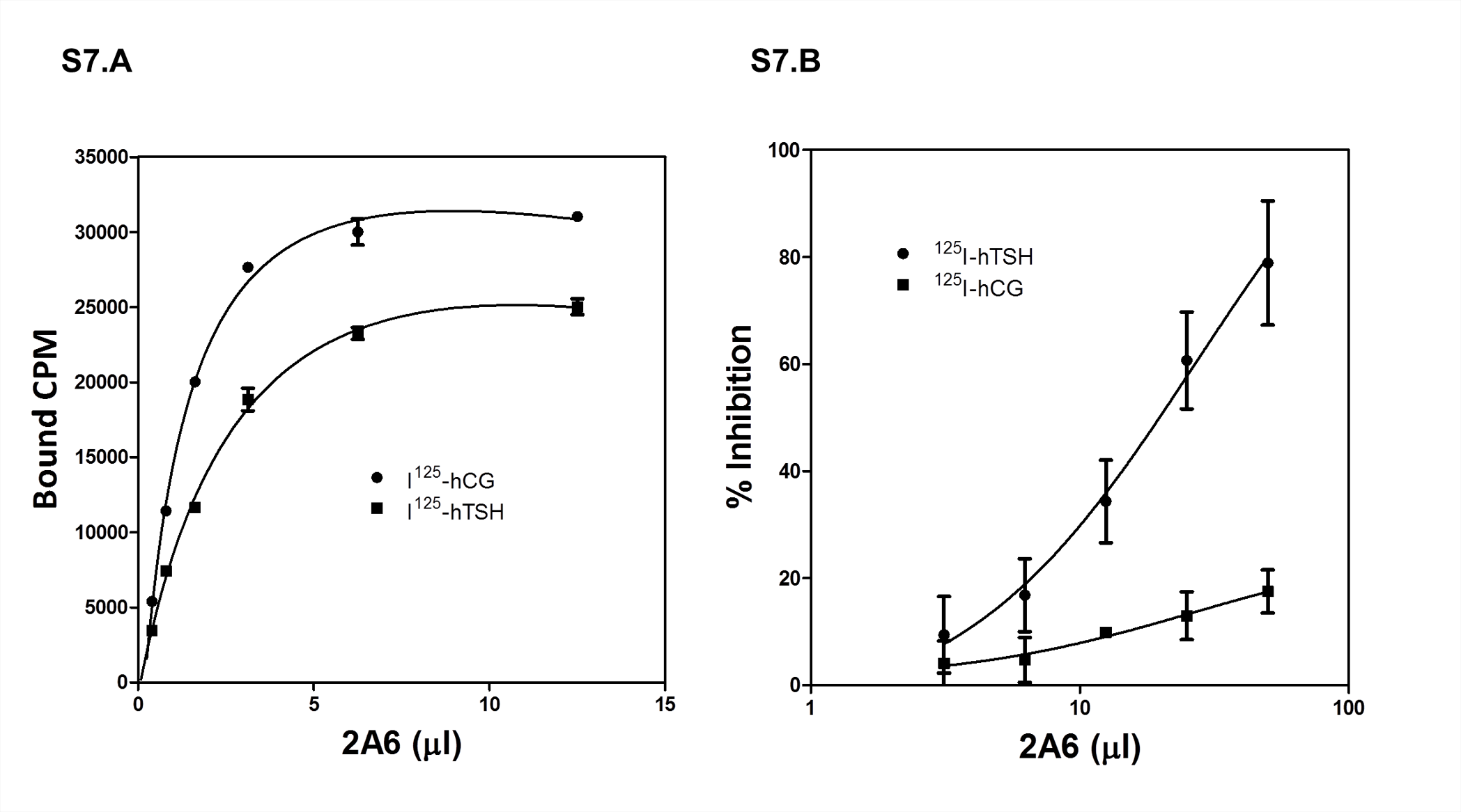

Supplement: Figure S7 — Characterization of α-subunit specific monoclonal antibody 2A6. S7A. Binding of 2A6 to labelled hCG/hTSH. 0.1–0.2 µCi (∼100,000 cpm) of 125I-hCG/125I-hTSH were incubated overnight with increasing dilutions of 2A6 antibody at room temperature (28–30°C). The antigen–antibody complexes were precipitated by centrifugal separation at 4000 g after the addition of goat- anti-mouse IgG and PEG-6000.The supernatant was discarded and the radioactivity in the pellet was counted using Perkin-Elmer auto gamma counter S7B. Effect of 2A6 on binding hCG/hTSH to their cognate receptor Effect of 2A6 on hormone–receptor interaction was determined by incubating the increasing dilution of 2A6 with either 125I-hCG or 125I-hTSH for 1 h at room temperature followed by addition of membrane preparation of the HEK293-LHR or HEK293-TSHR respectively and continuing the incubation for additional 1 h. labelled-hormone bound to the receptor was pelleted by centrifugation at 4000×g and counted in a gamma counter. Specific binding was obtained by subtracting the bound counts from non-specific binding obtained by addition of 0.5 µg of unlabelled hormone to each reaction mixture. Percentage ratio between difference of counts in presence and absence of 2A6 to the specific binding denoted the % inhibition of receptor binding by 2A6. (TIF) [file pone.0040291.s007.tif]

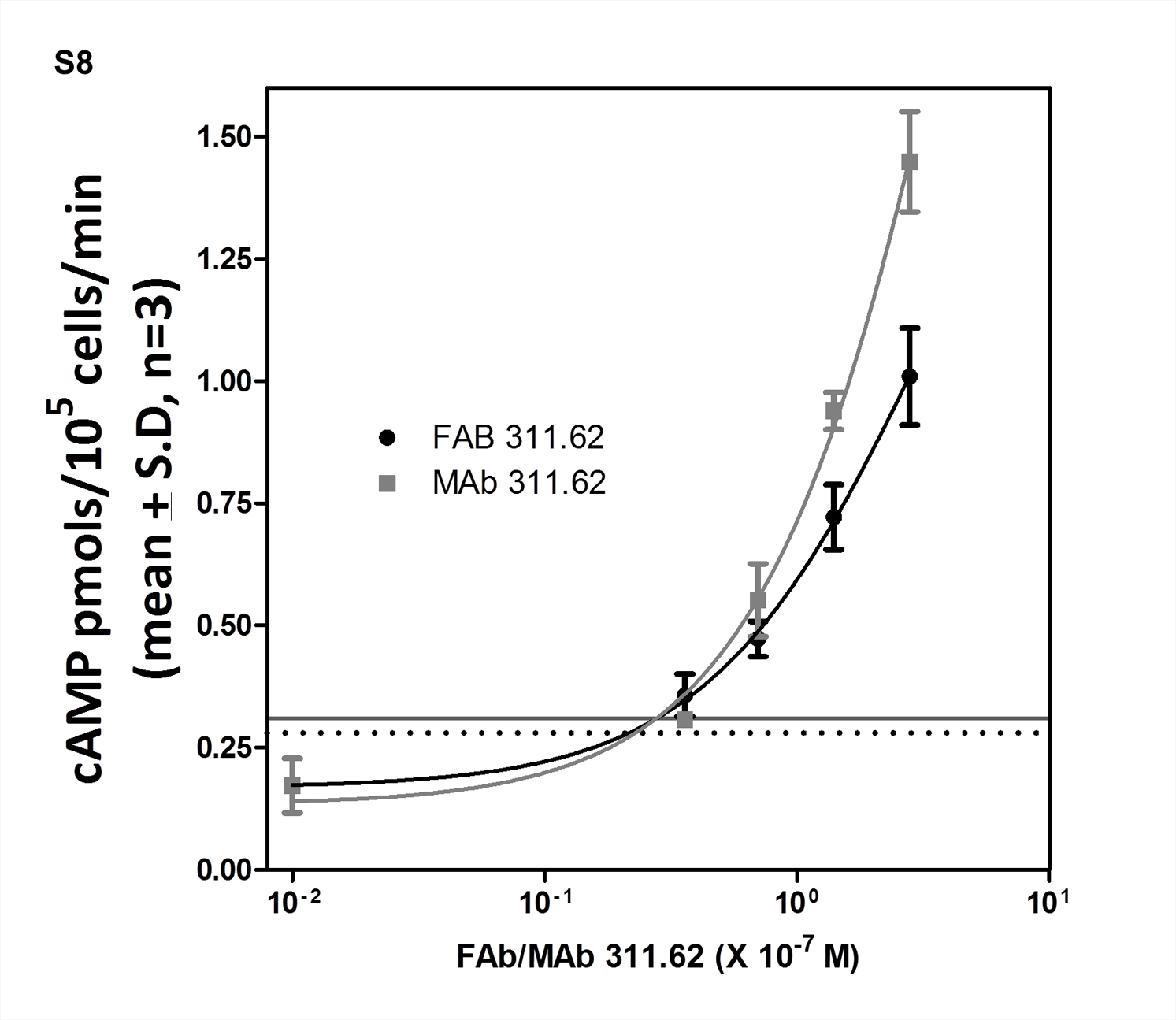

Supplement: Figure S8 — Stimulation of HEk292-hTSHR cells by FAb prepared from MAb 311.62 IgG. HEK293-hTSHR cells were incubated with increasing concentration of MAb 311.62 IgG or FAb fragments prepared from the in the absence of hTSH for 1 h at 37°C, and cAMP produced was determined by RIA. The solid and the dotted lines represent the cAMP produced by HEK293-hTSHR in presence of saturating concentrations (1 µM) of NMIgG or FAb prepared from NMIgG. (TIF) [file pone.0040291.s008.tif]

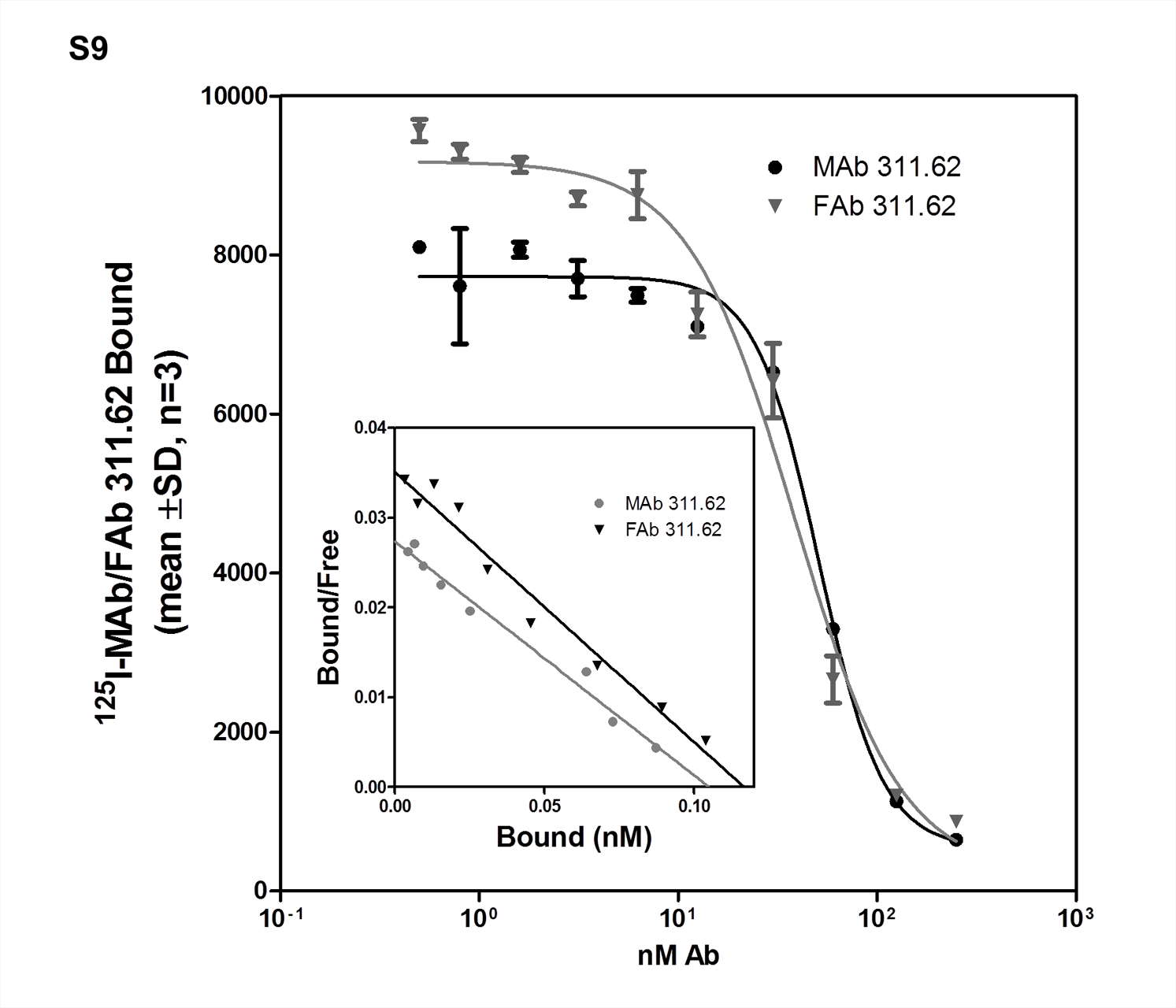

Supplement: Figure S9 — Receptor binding of radioiodinated FAB and MAb. HEK293-TSHR membrane preparation (10 µg) was incubated with different concentrations of FAb/Mab 311.62 in presence of 10 nM of 125I-FAb/MAb (specific activity tracer - 0.10 µci/fmol) at 37°C for 2 h in a reaction volume of 250 µl. The receptor bound radioactivity was centrifugally separated (4000 g at 4°C for 20 minutes) after addition of 2.5% PEG 6000 and counted in Perkin Elmer γ-counter. The non-specific binding was determined in presence of excess unlabeled antibody(10 µg/ml). (TIF) [file pone.0040291.s009.tif]

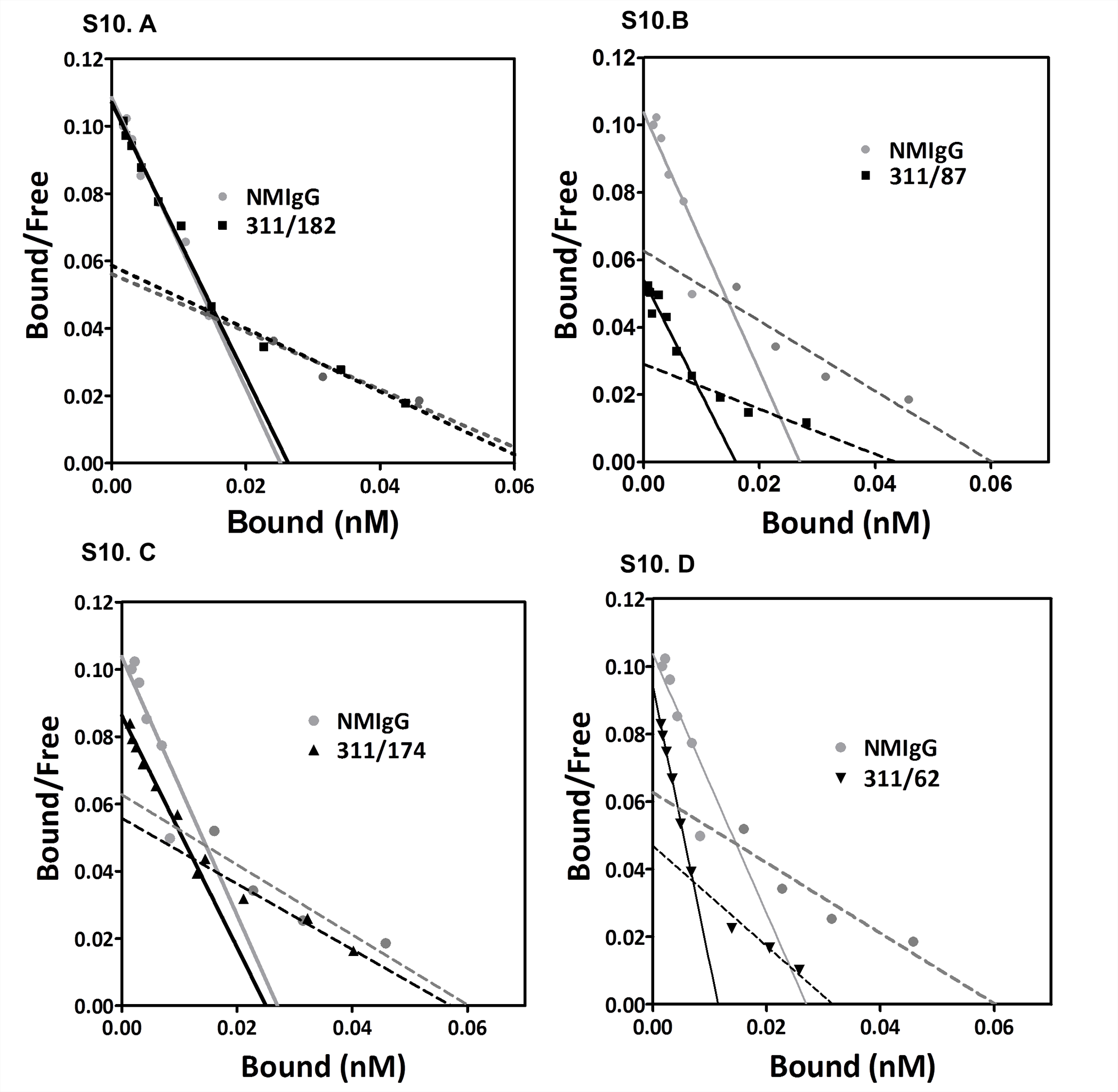

Supplement: Figure S10 — Scatchard Analysis of TSH-TSHR binding at excess TSH concentrations in presence of antibodies. 125I-hTSH (20000 CPM) was incubated with hTSHR membranes (20 µg/ml) with increasing concentrations (upto 1 µg/ml) of the unlabelled hTSH in the absence or presence of 50 µg/ml of A. 311.82 IgG, B. 311.87 IgG, C. 311.174 IgG or D. 311.62 IgG and the binding data converted into Scatchard plots. Two linear regression was resolved from the curvilinear Scatchard plot as described by De meyts etal, 1975. Solid lines denote the high affinity receptor component (apparent affinity, Kd1 = 0.11 nM in presence of NMIgG) whereas the broken lines represent low affinity receptor component (apparent affinity, Kd2 = 9.8 nM in presence of NMIgG) (TIF) [file pone.0040291.s010.tif]
