# Supplementary material for: The Hinge Region of Human Thyroid-Stimulating Hormone (TSH) Receptor Operates as a Tunable Switch between Hormone Binding and Receptor Activation
Source: PLoS One. 2012 Jul 6;7(7):e40291. doi: 10.1371/journal.pone.0040291 (PMC3391290; doi:10.1371/journal.pone.0040291)
Supplement: Table S1 — Isotyping of TSHR MAb. 1 µg/ml of IgG from subclones of each TSHR MAb was coated on polystyrene plates (Nunc Immunsorb) and indirect ELISA was carried out using mouse isotype specific antibodies (Mouse Monoclonal Antibody Isotyping Reagents, Sigma-Aldrich) as per the manufacturer’s specification. The Light chain subtype was determined by a mouse monoclonal antibody isotyping kit (dipstick format, GIBCO, BRL). (DOC) [file pone.0040291.s011.doc]

| **Table S1. Isotyping of TSHR MAb** | | | |
| --- | --- | --- | --- |
| TSHR MAb | Immunoglobin Isotype | Subclass | Light chain type |
| 311.62 | IgG | IgG1 | κ |
| 311.87 | IgG | IgG2a | κ |
| 311.174 | IgG | IgG3 | κ |
| 311.82 | IgG | IgG1 | κ |
| 413.1.F7 | IgG | IgG1 | κ |
| 1 µg/ml of IgG from subclones of each TSHR MAb was coated on polystrene plates (Nunc Immunsorb) and indirect ELISA was carried out using mouse isotype specific antibodies (Mouse Monoclonal Antibody Isotyping Reagents, Sigma-Aldrich) as per the manufacturer’s specification. The Light chain subtype was determined by a mouse monoclonal antibody isotyping kit (dipstick format, GIBCO, BRL). | | | |
